# Wheat *EARLY FLOWERING 3* affects heading date without disrupting circadian oscillations

**DOI:** 10.1093/plphys/kiac544

**Published:** 2022-12-01

**Authors:** Lukas Wittern, Gareth Steed, Laura J Taylor, Dora Cano Ramirez, Gabriela Pingarron-Cardenas, Keith Gardner, Andy Greenland, Matthew A Hannah, Alex A R Webb

**Affiliations:** Department of Plant Sciences, University of Cambridge, Downing Street, Cambridge CB2 3EA, UK; Department of Plant Sciences, University of Cambridge, Downing Street, Cambridge CB2 3EA, UK; Department of Plant Sciences, University of Cambridge, Downing Street, Cambridge CB2 3EA, UK; Department of Plant Sciences, University of Cambridge, Downing Street, Cambridge CB2 3EA, UK; Department of Plant Sciences, University of Cambridge, Downing Street, Cambridge CB2 3EA, UK; The John Bingham Laboratory, NIAB, 93 Lawrence Weaver Road, Cambridge, CB3 0LE, UK; The John Bingham Laboratory, NIAB, 93 Lawrence Weaver Road, Cambridge, CB3 0LE, UK; BASF, BBCC – Innovation Center Gent, Technologiepark-Zwijnaarde 101, 9052 Gent, Belgium; Department of Plant Sciences, University of Cambridge, Downing Street, Cambridge CB2 3EA, UK

## Abstract

Plant breeders have indirectly selected for variation at circadian-associated *loci* in many of the world's major crops, when breeding to increase yield and improve crop performance. Using an eight-parent Multiparent Advanced Generation Inter-Cross (MAGIC) population, we investigated how variation in circadian clock-associated genes contributes to the regulation of heading date in UK and European winter wheat (*Triticum aestivum*) varieties. We identified homoeologues of *EARLY FLOWERING 3* (*ELF3*) as candidates for the *Earliness* per se (*Eps*) *D1* and *B1 loci* under field conditions. We then confirmed a single-nucleotide polymorphism within the coding region of *TaELF3-B1* as a candidate polymorphism underlying the *Eps-B1 locus.* We found that a reported deletion at the *Eps-D1 locus* encompassing *TaELF3-D1* is, instead, an allele that lies within an introgression region containing an inversion relative to the Chinese Spring D genome. Using *Triticum turgidum* cv. *Kronos* carrying loss-of-function alleles of *TtELF3*, we showed that *ELF3* regulates heading, with loss of a single *ELF3* homoeologue sufficient to alter heading date. These studies demonstrated that *ELF3* forms part of the circadian oscillator; however, the loss of all homoeologues was required to affect circadian rhythms. Similarly, loss of functional *LUX ARRHYTHMO* (*LUX*) in *T. aestivum*, an orthologue of a protein partner of Arabidopsis (*Arabidopsis thaliana*) ELF3, severely disrupted circadian rhythms. *ELF3* and *LUX* transcripts are not co-expressed at dusk, suggesting that the structure of the wheat circadian oscillator might differ from that of Arabidopsis. Our demonstration that alterations to *ELF3* homoeologues can affect heading date separately from effects on the circadian oscillator suggests a role for *ELF3* in cereal photoperiodic responses that could be selected for without pleiotropic deleterious alterations to circadian rhythms.

## Teaser

The flowering time of a cereal is regulated by a circadian oscillator component separate from its function in the circadian system.

## Introduction

Circadian clocks are endogenous timing mechanisms with a near 24 h period that sequence biological events, provide anticipation of environmental rhythms, and permit measurement of daylength to regulate seasonal adjustments (McClung, 2019). Plant breeders have indirectly selected for variation at circadian-associated *loci* in many of the world's major crops when breeding to increase yield and improve crop performance by adapting genotypes to the local environment (Steed et al., 2021). Here, we investigated whether variation at circadian-associated *loci* contributes to wheat (*Triticum aestivum*) yield traits in the field using a Multiparent Advanced Generation Inter-Cross (MAGIC) mapping population (Mackay et al., 2014). Wheat is a major crop, providing approximately 20% of the dietary calories and protein for the world's population (Hawkesford et al., 2013). Since the green revolution, wheat yields have continued to increase but with the world's population estimated to reach 9.6 billion in 2050 (Gerland et al., 2014), it is likely that demand will outpace predicted yield increases (Hawkesford et al., 2013). Identification of genetic variation contributing to yield is critical to increasing the rate of yield gains in wheat.

Understanding of the nature of plant circadian oscillators is based primarily on the investigation of Arabidopsis (*Arabidopsis thaliana*). In that model, the circadian oscillator contains a series of interlocking transcriptional feedback loops. The circadian oscillator cycle can be considered to start at dawn when expression of the transcriptional repressors CIRCADIAN CLOCK ASSOCIATED 1 (CCA1) and LATE ELONGATED HYPOCOTYL (LHY) peaks, followed sequentially by peaks of expression of *PSEUDO RESPONSE REGULATOR 9* (*PRR9*)*, PRR7, PRR5, PRR3,* and finally, *PRR1* (also known as *TIMING OF CAB EXPRESSION 1* (*TOC1*)) at dusk (Hsu and Harmer, 2014). PRR9 and PRR7 repress the expression of *CCA1* and *LHY* which releases repression of evening phased genes including, *TOC1, GIGANTEA* (*GI*)*, EARLY FLOWERING 3* (*ELF3*)*, ELF4,* and *LUX ARRHYTHMO* (*LUX*) (Hsu and Harmer, 2014). LUX is a MYB-like transcription factor that associates with ELF3 and ELF4 to form the tripartite evening complex (EC), which represses the expression of *GI, TOC1, PRR7, PRR9,* and *LUX* (Dixon et al., 2011; Nusinow et al., 2011; Herrero et al., 2012). Mutation of any of the three EC components causes arrhythmia of the Arabidopsis circadian oscillator in continuous light and temperature (Nagel and Kay 2012).

Orthologues of *ELF3* underlie *loci* regulating heading date in multiple crops, including barley (*Hordeum vulgare*) early *maturity8* (*eam8*) (Faure et al., 2012), rice (*Oryza sativa*) *heading date17* (*Hd17*) (Matsubara et al., 2012), Earliness *per se* (*Eps*)-*A^m^1* in *Triticum monococcum* and *Eps-D1* in *T. aestivum* (Zikhali et al., 2016). In hexaploid wheat, the magnitude of the *Eps-D1* effect on the heading date increases with decreasing temperature (Ochagavía et al., 2019). Orthologues of *TOC1* are associated with heading date, plant height, and thousand-grain weight (Sun et al., 2020). An orthologue of *LUX* is a candidate for *Eps-3A^m^* in *T. monococcum* (Gawronski et al., 2014) and *HvLUX* is a candidate gene for *eam10* in barley (Campoli et al., 2013). Furthermore, the *Photoperiod-1* (*Ppd-1*) genes, which contribute to photoperiodic regulation of ear emergence are orthologues of Arabidopsis *PRRs* (Beales et al., 2007; Shaw et al., 2012; Bentley et al., 2013; Turner et al., 2013).

We used the allelic diversity of a wheat MAGIC population to investigate the role of circadian clock genes in regulating yield component traits in the field. The parent varieties for the MAGIC population used in this study (Alchemy, Brompton, Claire, Hereward, Rialto, Robigus, Soissons, and Xi-19) are representative of the United Kingdom and European winter wheat varieties, capturing >80% of the polymorphisms found across the UK wheat varieties from the last 70 years (Gardner et al., 2016). Our data demonstrate that modified *ELF3* function can affect the regulation of flowering time without disrupting circadian oscillations and thus potentially avoid associated yield penalties (Dodd et al., 2005). Together, this suggests that *ELF3* is a possible breeding target for crop improvement. We find evidence that in wheat, *ELF3* is expressed in the morning, rather than the evening as it is in Arabidopsis and conclude there are likely differences in the role of *ELF3* in the circadian system between wheat and Arabidopsis. Our finding of separable roles for *ELF3* in the circadian oscillator and the regulation of heading date suggest a direct role for ELF3 in photoperiodic regulation in cereals.

## Results

### Earliness *per se loci Eps-B1* and *Eps-D1* segregate in an eight-parent wheat MAGIC population

**Figure 1:**
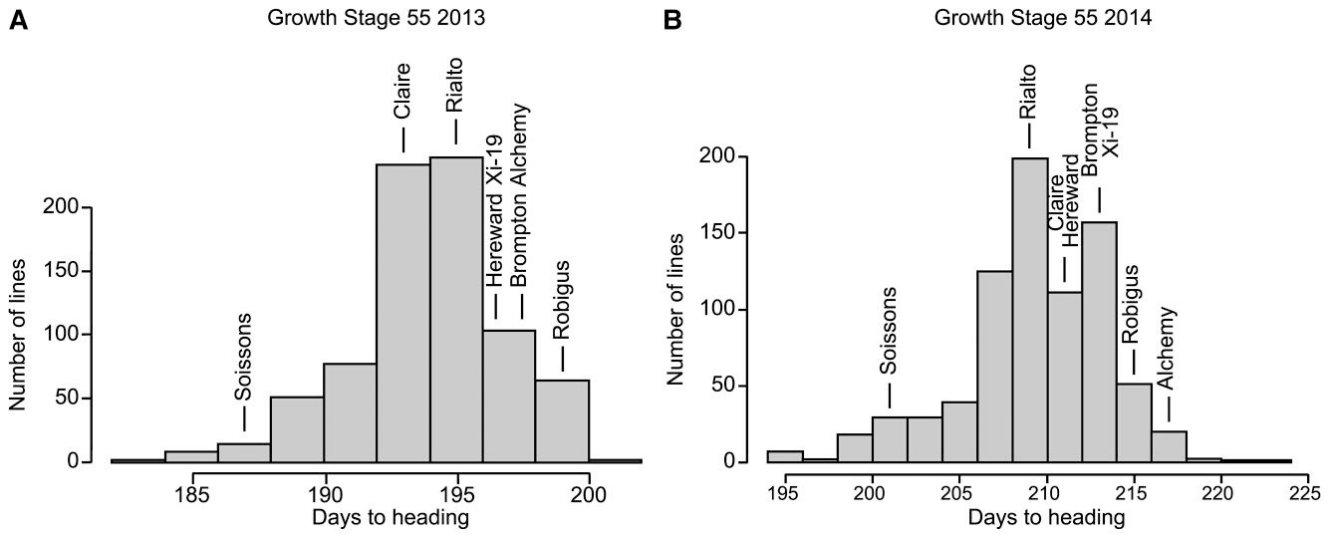
Variation in time to reach growth stage 55 across a United Kingdom wheat MAGIC population. Time in days for individual MAGIC recombinant inbred lines (RILs) to reach growth stage 55 (GS55) for the (A) 2012/2013 growing season and (B) 2013/2014 growing season. The number of days after sowing to reach GS55 for the eight MAGIC parents is indicated.

We performed quantitative trait locus (QTL) analysis on a set of replicated trial data for 784 recombinant inbred lines (RILs) for the National Institute of Agricultural Botany (NIAB) Elite MAGIC population from the 2012/2013 and 2013/2014 growing seasons at the NIAB experimental farms in Cambridge, UK. Growth stage 55 (GS55, equivalent to heading date) (Zadoks et al., 1974) was chosen for analysis and QTL mapping to enable comparisons with previous studies (Griffiths et al., 2009). In both 2013 and 2014, Soissons, which contains the *Ppd-D1a* (photoperiod insensitive) allele, reached GS55 6–8 days earlier than any of the other MAGIC parents, which contain the *Ppd-D1b* allele (photoperiod sensitive) (Figure 1). Heading date of the other MAGIC parents varied over 5.5 days in 2013 and 7 days in 2014.

We performed the QTL analysis using MPWGAIM (Verbyla et al., 2014) for GS55 phenotypes in both years and tested whether any circadian clock genes co-locate with the identified QTLs (<2 cM to the nearest 90 k marker or <5 Mb to the nearest gene). Because we are interested in the effects of the circadian clock on yield traits, we report only QTLs associated with circadian clock genes. We found that four circadian gene orthologues (*TaELF3-B1, TaELF3-D1, Ppd-1, TaZTL-7A*) fell within 0.20, 0.27, 0.58, and 4.31 Mb, respectively, of one of the QTL flanking markers (Table 1). The absence of significant heading date QTLs linked to vernalization genes such as *VRN1* confirmed that complete vernalization had occurred, ensuring that observed variations in heading date were true Earliness *per-se* (*Eps*) effects rather than a variation in the vernalization requirements between the MAGIC parents (Bentley et al., 2013; Camargo et al., 2016). A previous study identified a single GS55 QTL in the NIAB eight-parent MAGIC population (Camargo et al., 2016) but this difference in findings is likely a result of different growth conditions (greenhouse compared to field), lower replicate number (208 RILs in pots compared to 784 RILs in field plots) and the use of a different mapping methodology. Further validation of our analysis pipeline and approaches are described in the methods and reported in Supplemental Table 1.

**Table 1:** Summary of circadian clock-associated QTLs and their mapping positions. Output from univariate QTL model for the NIAB 2013 and 2014 GS55 data. Gene (Mb) refers to the BLAST hit starting coordinate of the gene's cDNA against the IWGSCRefSeqv1.1. Dist (cM) refers to the marker’s genetic mapping distance in centiMorgans. Dist (Mb) refers to the BLAST hit starting coordinate of the marker sequence against the IWGSCRefSeqv1.1. LOGP is −log10(*P*) which corresponds to the overall significance of the QTLs as a measure of its strength. % var is the percentage of genetic variance contributed by the QTL. Further description of the methodology and column headers can be found in (Verbyla et al., 2014).

Circadian clock gene	Gene (Mb)	Chr	L. Marker	dist (cM)	dist (Mb)	R. Marker	dist (cM)	dist (Mb)	Year	LOGP	% var
***TaELF3***	685,646,575	1B	Ra_c109187_371	296.34	685,741,261	RAC875_c37075_283	297	687,715,336	2013	4.48	3.4
		1B	Ra_c109187_371	296.34	685,741,261	RAC875_c37075_284	297	687,715,336	2014	3.2	2
***TaELF3***	493,485,612	1D	Kukri_c29687_369	100.95	493,212,138	Kukri_rep_c69829_307	101.95	488,577,079	2014	2.32	4.1
***Ppd-1***	33,952,591	2D	Kukri_c27309_590	48.57	31,806,310	BS00064538_51	56.64	33,370,535	2013	20.04	17.2
		2D	Kukri_c27309_590	48.57	31,806,310	BS00064538_51	56.64	33,370,535	2014	43.92	30.2
***TaZTL***	625,638,942	7A	Kukri_c6676_172	207.62	610,934,248	Ku_c2990_1939	208.12	621,322,194	2013	2.87	2.6

**Figure 2:**
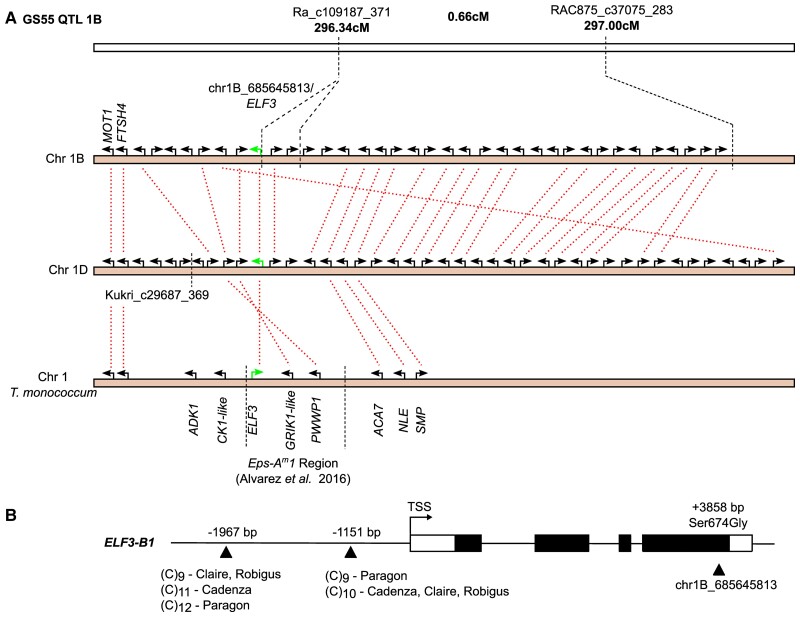
*EARLY FLOWERING 3* is a candidate gene for Earliness per se*-B1* (*Eps-B1*). (A) Syntenic relationships of the *Eps-B1* QTL to the *Eps-D1* quantitative trait locus (QTL) and the *T. monococcum Eps-A^m^1* QTL. *ELF3* highlighted in green. (B) *TaELF3-B1* gene model with exons represented by filled rectangles, untranslated regions (UTRs) as white rectangles. The two promoter cytosine repeat polymorphisms with a location relative to the transcription start site (TSS) are indicated with the cytosine (C) repeat number for Claire, Robigus, Cadenza, and Paragon. The Ser674Gly single-nucleotide polymorphism (SNP) is situated within exon 4 of *ELF3-B1*.

A QTL on the distal end of chromosome 1B, delineated by the markers Ra_c109187_371 and RAC875_c37075_283, is mapped closely to the physical position of *TaELF3-B1* (TraesCS1B02G477400) (Table 1). The 1B locus also had a homoeologous QTL on chromosome 1D delineated by the markers Kukri_c29687_369 and Kukri_rep_c69829_307 identifying *TaELF3-D1* (TraesCS1D02G451200) as a candidate gene (Table 1, Figure 2A). These QTLs co-locate with the previously described heading date QTLs *Eps-B1* and *Eps-D1* (Griffiths et al., 2009). In our data, *Eps-B1* had a significant effect in both years, explaining 3.4% of genetic variance in GS55 in 2013 and 2% in 2014 (Table 1). *Eps-D1* was also detected in both years but only had an overall significant effect (−log10(*P*) = 2.32) in 2014, accounting for 4.1% of genetic variance in GS55 in that year (Table 1). The *Eps-B1* and *Eps-D1 loci* were chosen for further in-depth analysis because of their proximity to *ELF3* homoeologues.

### *TaELF3-B1* is a candidate gene for the *Eps-B1* QTL

At the 1B QTL, the alleles derived from the MAGIC parents Soissons, Alchemy, Robigus, and Claire are associated with a relative reduction in the time to GS55, indicated by the negative sign of the QTL, while alleles from Rialto, Hereward, Xi-19, and Brompton are associated with a delay to GS55, indicated by the positive sign of the QTL (Table 2). The interval between the markers Ra_c109187_371 (296.34 cM/685.7 Mb) and RAC875_c37075_283 (297.00 cM/687.7 Mb) is 0.66 cM, corresponding to a physical length of ca. 2 Mb (Figure 2A).

**Table 2:** 2013 and 2014 GS55 QTL summary for the eps-B1 locus. Estimated parental haplotype effects on recombinant inbred line (RIL) best linear unbiased predictors (BLUPs). Abbreviations as per (Verbyla et al., 2014): Dist (cM) refers to the marker’s genetic mapping distance in centiMorgans (cM). LOGP is −log10(*P*) and corresponds to the overall significance of the QTL as a measure of its strength. Founder Prob: probability QTL is from founder shown (*P*). Strength of associations can thus be reported at the overall or founder level. Size: Size of the effect of each founder allele (days to GS55).

QTL	Year	Founder	Size	Founder Prob	% var	LOGP	KASP (chr1B:685645813)
***Eps-B1***	2013	Alchemy	−0.555	0.054	3.4	4.48	A:A
		Brompton	0.317	0.123			G:G
		Claire	−0.076	0.415			A;A
		Hereward	0.623	0.008			G:G
		Rialto	0.331	0.118			G:G
		Robigus	−0.102	0.376			A:A
		Soissons	−0.683	0.003			A:A
		Xi-19	0.053	0.426			G:G
***Eps-B1***	2014	Alchemy	−0.448	0.196	2	3.2	A:A
		Brompton	0.395	0.178			G:G
		Claire	−0.442	0.202			A:A
		Hereward	0.591	0.072			G:G
		Rialto	0.39	0.182			G:G
		Robigus	−0.313	0.264			A:A
		Soissons	−1.085	0.002			A:A
		Xi-19	0.453	0.15			G:G

The IWGSCv1.1 gene annotation suggests there are 26 high-confidence gene models within the interval of Ra_c109187_371 and RAC875_c37075_283. Based on current gene annotation and orthology to cloned rice genes (Yao et al., 2018), there are no candidates for the *Eps-B1* locus in addition to those described previously (Faricelli et al., 2010; Alvarez et al., 2016; Zikhali et al., 2016). Compared to the *T. monococcum* physical map of the *Eps-A^m^1* region (Alvarez et al., 2016), *TmADK1* (*ADENYLATE KINASE 1,* similar to rice putative kinase *ADK1)* and *TmCK1-like* (*CASEIN KINASE I ISOFORM DELTA-LIKE)* are not syntenic and the locus containing *TaELF3*, *TaGRIK1-like* (*GEMINIVIRUS REP INTERACTING KINASE 1 LIKE*) and *TaPWWP1* (*PWWP domain-containing protein*) is inverted (Figure 2A). Nineteen genes had homoeologues on the syntenic 1D chromosome (Figure 2A).

Publicly available sequences for *TaELF3-B1* in Claire, Robigus (earlier heading alleles) (Clavijo et al., 2017) and Rialto and Cadenza—the parents of Xi-19—(later heading alleles) (Zikhali et al., 2016) differed by one nonsynonymous SNP (single-nucleotide polymorphism) within exon 4 at position chr1B:685645813. No further nonsynonymous SNPs were identified from the 820k SNP dataset from CerealsDB (Wilkinson et al., 2012), which contains all MAGIC parents.

The SNP (Adenine at position 2020 of CDS to Guanine; A2020G) causes a predicted deleterious amino acid substitution from the ancestral serine to a glycine residue at position 674 of the predicted primary amino acid sequence (Ser674Gly; SIFT 0.01) (Figure 2B). A complete allele-specific PCR (KASP) marker was designed for this SNP and used to genotype the MAGIC population. The KASP marker mapped to the same genetic position as Ra_c109187_371 and segregated with the predicted founder effects, confirming that *TaELF3-B1* is a part of the 1B GS55 QTL.

This predicted glycine at residue 674 of *TaELF3-B1*, associated with late heading, is globally rare in wheat. In the 1000Exome data (He et al., 2019), 4.87% of lines have the predicted glycine residue, and in the wheat HapMap panel (Jordan et al., 2015) only Rialto has the predicted glycine residue out of the 62 globally diverse lines analyzed (Supplemental Table 2). Similarly, of the 14 hexaploid varieties currently available from the 10+ Wheat Genome project, only three (Cadenza, Paragon, ArinaLrFor) possess the non-ancestral glycine residue (Supplemental Table 2). However, analysis of multiple datasets (see methods) found that the non-ancestral glycine674 is more common in wheat varieties with a UK pedigree (9/13 varieties) (Supplemental Table 3). The alignment of *TaELF3* orthologues shows that the predicted serine residue is also highly conserved across monocots (Supplemental Figure 1).

When looking for other potentially candidate sequence variations, we found that within the *ELF3-B1* promoter regions of Claire, Robigus, Paragon, and Cadenza, there are two sites polymorphic for the number of repeated cytosines ((C)_n_) located 1967 bp and 1151 bp upstream of the transcriptional start site (TSS) (Figure 2B). Variation in CT repeat number within the 5′-UTR region of the wheat *cellulose synthase like* (*Csl*) gene is associated with *Csl* expression and tiller number (Hyles et al., 2017). As Robigus and Claire have an early heading phenotype at the *Eps-B1* locus and Cadenza, a late heading phenotype (Alvarez et al., 2016), it is unlikely that the promoter (C)_n_ polymorphisms cause the changes in heading date because there is only a difference of two cytosines at the 1967bp (C)_n_ site between Cadenza and Robigus/Claire (Figure 2B). This conclusion is supported by our finding that there is no correlation between the (C)_n_ genotype and *TaELF3* expression (ANOVA F = 0.484, *P* = 0.748) (Supplemental Figure 2). It was not possible to quantify the relative transcript abundance of the *TaELF3-B1* homoeologue due to a lack of homoeologous SNPs in the coding region of *TaELF3* as previously reported (Zikhali et al., 2016). Furthermore, *TaELF3-B1* in Julius, Lancer, Mace, Norin61, and Sy Mattis seems to be inverted compared to ArinaLrFor, CDC Landmark, CDC Stanley, Chinese Spring, and Jagger. However, as none of these lines share the same haplotype with ArinaLrFor and Paragon (which both carry the Ser674Gly SNP) at *ELF3-B1,* it is unlikely that an inversion is the cause behind the *Eps-B1* QTL. Thus, we conclude that the Ser674Gly SNP in *ELF3-B1* is the best candidate to underlie *Eps-B1*.

### Subtelomeric introgression and inversion might underlie the *Eps-D1* heading date QTL

A subtelomeric deletion in Cadenza and Spark was previously described as the causal polymorphism underlying the *Eps-D1* locus using Spark × Rialto, Avalon × Cadenza mapping populations (Zikhali et al., 2016; Ochagavía et al., 2019). Our QTL analysis for 2012 (Supplemental Table 1) and 2014 (Table 1) shows a minor QTL overlapping this locus. In both years, the Xi-19 founder genotype is associated with earlier heading (2012: −1.26 days; *P* = 0.015; 2014; −0.79 days; *P* = 0.053) (Table 3). However, correct genetic map construction and resulting assignment of founder genotypes at this locus might be more difficult, given the high-density marker region previously identified at the end of 1D. These high-density marker regions were hypothesized to be associated with introgressions (Gardner et al., 2016). Based on the previously reported deletion in Cadenza and Spark (Zikhali et al., 2016) and the pedigree of Xi-19 (Cadenza/Rialto//Cadenza), we hypothesized that the Cadenza allele inherited from Tonic was a likely candidate for the observed QTL effects. By analyzing data from the recently published wheat assemblies (Walkowiak et al., 2020), we suggest that polymorphism is due to a subtelomeric inversion derived from introgressed material rather than a deletion. A BLAST search of the Chinese Spring *TaELF3-D1* genomic sequence against the 10+ Wheat Genome Project varieties identified high-confidence matches in all varieties including Cadenza. Interestingly, the sequence retrieved from the variety Jagger was identical to Cadenza. However, the sequence identity of the Jagger and Cadenza coding sequence for TraesJAG1D01G492900.1 and TraesCAD_scaffold_026352_01G000400.1 compared to the Chinese Spring gene is much lower (96.72%) than for the remaining 1D coding sequences (>99.9%). The Jagger and Cadenza 1D coding sequences have a greater similarity to the Chinese Spring *TaELF3-A1* (97.50%) and Chinese Spring *TaELF3-B1* (97.88%) homoeologues than to Chinese Spring *TaELF3-D1*.

**Table 3:** 2012 and 2014 GS55 QTL summary for the *Eps-D1* locus. Estimated parental haplotype effects on RIL BLUPs. Abbreviations as per (Verbyla et al., 2014): Dist (cM) refers to the marker’s genetic mapping distance in centiMorgans (cM). LOGP is −log10(*P*) and corresponds to the overall significance of the QTL as a measure of its strength. Founder Prob: probability QTL is from founder shown (*P*). Strength of associations can thus be reported at the overall or founder level. Size: Size of the effect of each founder allele (days to GS55).

QTL	Year	Founder	Size	Founder Prob	% var	LOGP	PCR (Figure 3D)
***Eps-D1***	2012	Alchemy	0.552	0.254	0.9	0.56	1097 bp
		Brompton	0.105	0.446			1097 bp
		Claire	−0.11	0.447			1097 bp
		Hereward	0.021	0.49			1097 bp
		Rialto	−0.044	0.477			1097 bp
		Robigus	0.003	0.498			1097 bp
		Soissons	0.734	0.134			1097 bp
		Xi-19	−1.261	0.015			776 bp
***Eps-D1***	2014	Alchemy	−0.058	0.472	4.1	2.32	1097 bp
		Brompton	0.869	0.104			1097 bp
		Claire	−1.412	0.051			1097 bp
		Hereward	−0.582	0.252			1097 bp
		Rialto	−0.071	0.457			1097 bp
		Robigus	−0.265	0.38			1097 bp
		Soissons	1.244	0.013			1097 bp
		Xi-19	−0.79	0.053			776 bp

Visualization of the 1D chromosome for the 10+ Wheat Genome Project varieties using the Crop Haplotypes viewer (Brinton et al., 2020) showed a shared haplotype block at the distal end of chromosome 1D between Cadenza (Haplotype viewer coordinates: 482.8–495.4 Mbp) and Jagger (480.0–493.5 Mbp), where *TaELF3*-*D1* is found.

**Figure 3:**
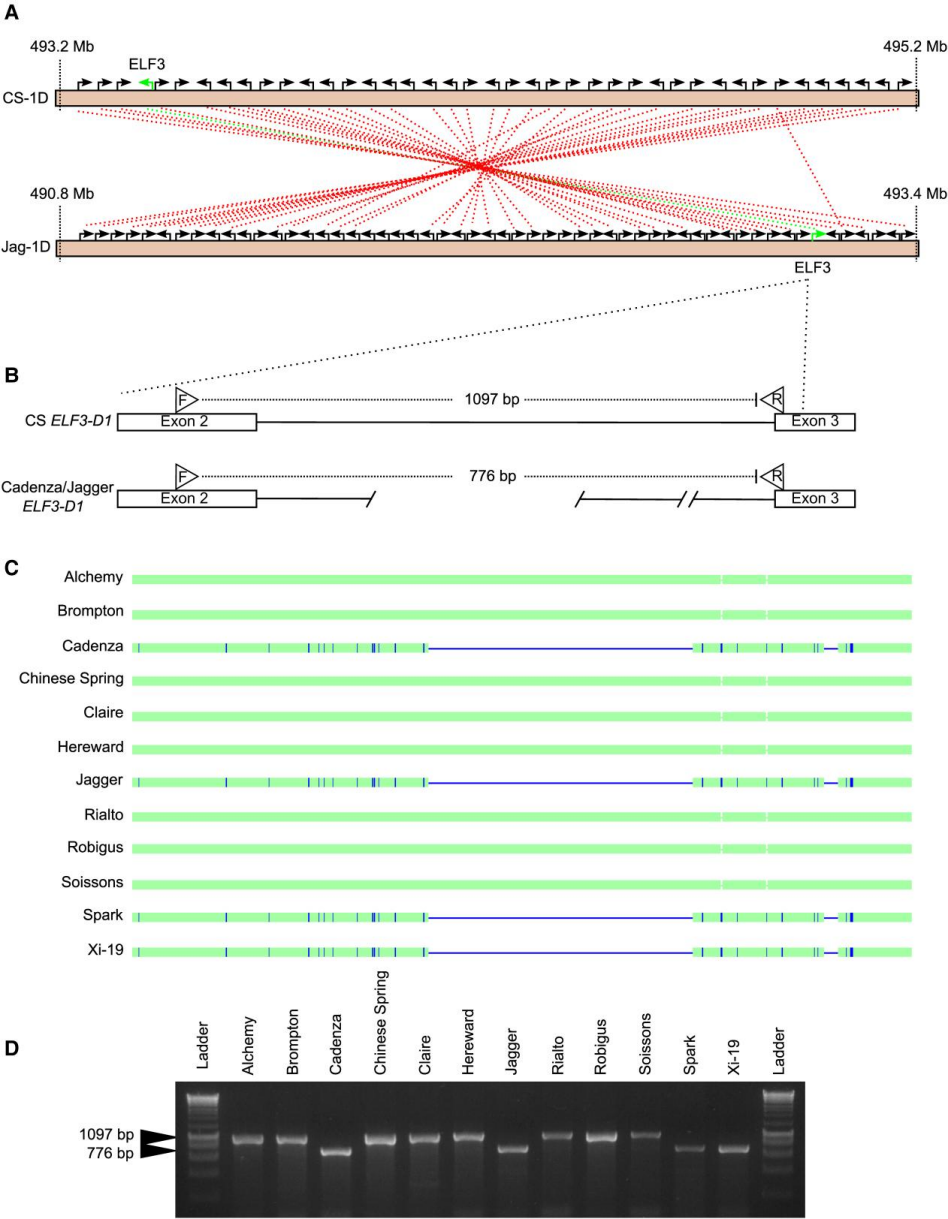
A subtelomeric chromosomal introgression and inversion containing *EARLY FLOWERING 3-D1* is likely to be the causal polymorphism underlying the *Earliness* per se*-D1* quantitative trait loci. (A) Syntenic relationships between the distal end of Chinese Spring (CS) 1D chromosome (IWGSCv1.1) and Jagger (Jag) 1D (PGSBv2.0), *TaELF3*-*D1,* labelled "ELF3" and highlighted in green, whereas as each unlabelled black arrow corresponds to a gene in the forward (pointing right) or reverse (pointing left) strand. (B) the Cadenza/Jagger *ELF3-D1* contains a deletion within intron 2 which is not present in CS, F, and R, referring to relative primer binding sites for PCR amplification shown in (C). (D) Agarose gel separation of polymerase chain reaction (PCR) products from (C). A PCR product of 776 base pairs (bp) is indicative of the presence of a subtelomeric chromosomal introgression and inversion.

The Cadenza v1.1 reference sequence has yet to be assembled into chromosomes, but since the Crop Haplotypes viewer (Brinton et al., 2020) data indicated identical by state (IBS) haplotypes between Jagger and Cadenza, we compared the distal end structure of the Jagger 1D chromosome with Chinese Spring. The end of the Jagger chromosome contains an inversion of approximately 2.6 Mbp relative to Chinese Spring which includes *TaELF3-D1* (Figure 3A). Alignment of the Jagger/Cadenza *TaELF3-D1* genomic sequence with Chinese Spring *TaELF3-D1* showed some sequence differences including a deletion within intron 2 (Figure 3B, Supplemental Table 4). We developed a homoeologue specific PCR assay detailed in Figure 3B that amplifies the region of *TaELF3-D1* containing intron 2 and used this as a simple marker for the 1D introgression. We confirmed that, of the eight MAGIC parents, only Xi-19 carried the Cadenza/Jagger *TaELF3-D1* allele (Figure 3, C and D). As hypothesized, Spark and Tonic also carry the Cadenza *TaELF3-D1* allele (Figure 3, C and D and Supplemental Figure 3) as well as the varieties Maris Fundin and Cordiale which show introgression signatures on the distal end of chromosome 1D (Scott et al., 2021). A simplified pedigree diagram shows how some of the cultivars are related (Supplemental Figure 4). The intron 2 deletion is also present in *Aegilops speltoides* and *Triticum timopheevii* (Supplemental Figure 5).

Given the low sequence conservation of the *TaELF3-D1* gene in Cadenza and Jagger and the presence of the intronic deletion in *Ae. speltoides* and *Triticum timopheevii*, we investigated the wider phylogeny of the 1D Jagger/Cadenza region using the 820k SNP dataset from CerealsDB. This showed that Cadenza, Xi-19, and Spark as well as several other Tonic descendants (KWS-Podium, Cordiale, Gallant, Duxford) and five other cultivars (Moisson, Bacanora, Tuerkis, Badger, Pavon) clustered separately from the remaining *T. aestivum* cultivars (Supplemental Figure 6). These lines also cluster with six Watkins lines from India (Watkins34), Yugoslavia (Watkins352), China (Watkins141), UK (Watkins103), Cyprus (Watkins292), and Turkey (Watkins299). *T. timopheevii* clusters closely with the above lines. Gene alignment of *ELF3-D1* orthologues and alignment of 300 kb surrounding *ELF3-D1* also supports an origin outside the D genome for the Jagger/Cadenza *ELF3-D1* gene (Supplemental Figure 7, Supplemental Table 5).

### Allelic variation in *TaELF3* had no detectable effect on circadian rhythms in the MAGIC parent varieties

**Figure 4:**
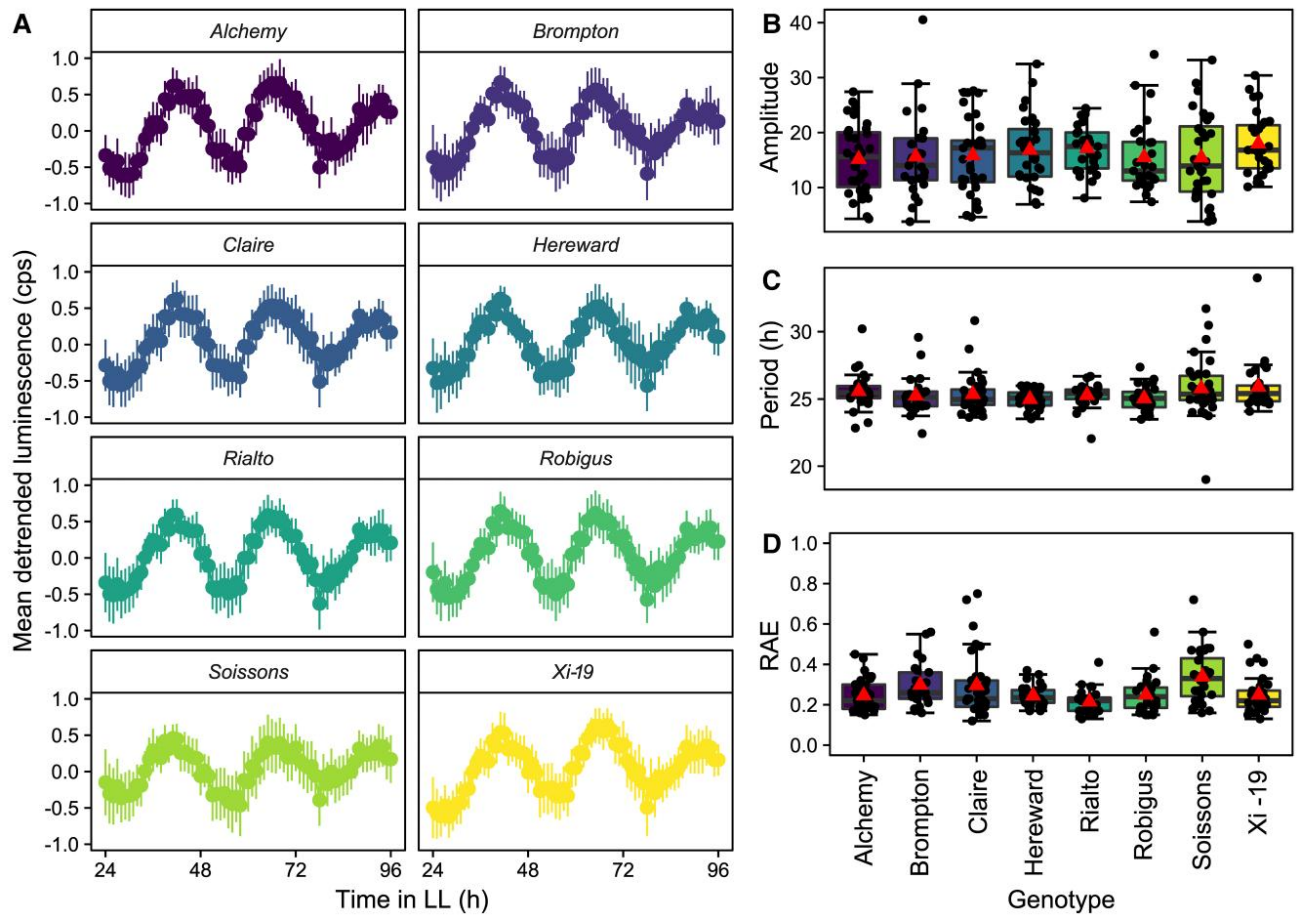
Circadian rhythms of delayed chlorophyll fluorescence in the MAGIC parents. (A) Mean delayed fluorescence (DF) luminescence in counts per second (cps) normalized to −1 to 1 using Biodare2 with error bars representing standard deviation (*n* = 27–33). Circadian amplitude (B), period (C), and relative amplitude error (RAE) (D) calculated using FFT-NLLS (Biodare2). Upper and lower hinges represent the first and third quartiles (25th and 75th percentiles), the middle hinge represents the median value, whiskers represent the third quartile + 1.5*interquartile range (IQR) and the first quartile – 1.5*IQR, red triangle represents the mean value, and black dots represent individual replicates. LL is constant light.

We investigated if the variations at *TaELF3-B1* and *TaELF3-D1* in the MAGIC parents, or other *loci,* might be associated with alterations of circadian rhythms in a manner that might explain the effects on heading date. We quantified circadian rhythms in the MAGIC parent varieties by measuring the period and amplitude of rhythms of delayed chlorophyll fluorescence (DF) in otherwise constant light (Gould et al., 2009). There were robust circadian rhythms of DF in all the MAGIC parent lines (relative amplitude error (RAE) < 0.5) (Figure 4, A and B). Hereward has the shortest circadian period (25.0 h) (Figure 4C) and Xi-19 the longest period (26.2 h) but the difference between these periods and the mean of all parents are not significant (*P* = 0.132) (Figure 4C). To determine whether the lack of an altered circadian phenotype in the MAGIC parent lines was the result of masking by compensatory alleles at other *loci,* we selected some of the earliest and latest heading MAGIC RILs from 2014 and used chlorophyll *a* fluorescence (PF) to measure circadian rhythms in continuous light (Supplemental Figure 8). There was no significant relationship between heading (early or late) and circadian period (*P* = 0.4216) (Supplemental Figure 8B) or between heading and circadian rhythm amplitude (*P* = 0.7385) (Supplemental Figure 8C). We detected robust rhythms of PF in all lines (RAE < 0.5) (Supplemental Figure 8D) indicating that in these lines, there was no effect on circadian rhythm robustness. We then investigated whether there was any significant relationship between circadian rhythm parameters and the *ELF3-B1, ELF3-D1,* or *Ppd-1* alleles associated with the altered heading date. There was no significant difference between the circadian period and *ELF3-B1* alleles (*P* = 0.542), *ELF3-D1* alleles (*P* = 0.521), or *Ppd-1* alleles (*P* = 0.507). Similarly, there was no significant difference between the circadian amplitude and *ELF3-B1* alleles (*P* = 0.942), *ELF3-D1* alleles (*P* = 0.1302), and *Ppd-1* alleles (*P* = 0.966). Thus, the allelic diversity in homoeologues of *TaELF3* that was associated with alterations in heading date did not cause detectable changes in circadian rhythms.

### *TtELF3* has separable effects on heading date and the wheat circadian clock

**Figure 5:**
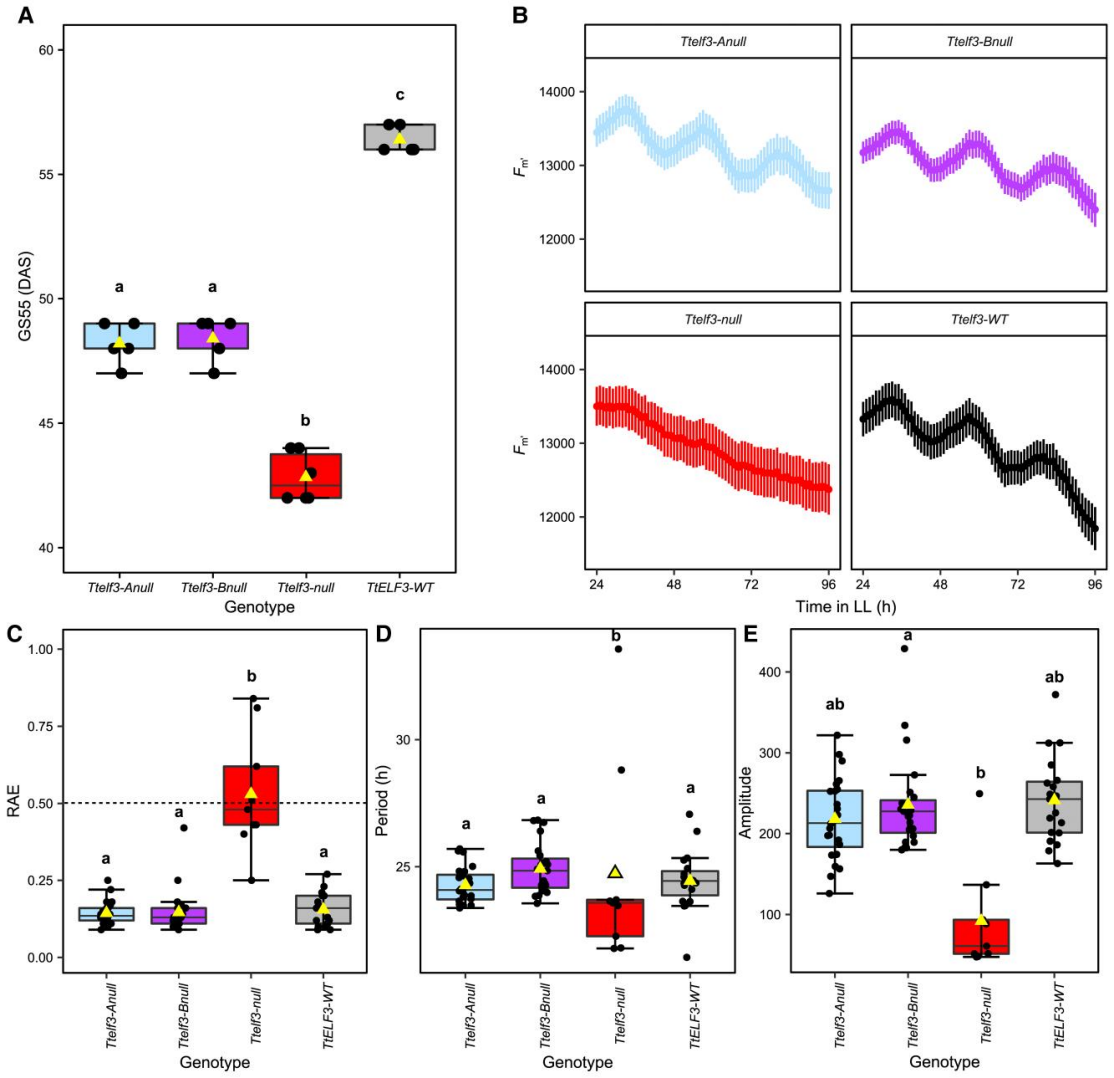
Mutation of single *ELF3* homoeologues affects heading date but not circadian rhythms of chlorophyll *a* fluorescence and *Ttelf3*-null is arrhythmic in continuous light. (A) GS55 for *Ttelf3-*null, *Ttelf3-*Anull, *Ttelf3-*Bnull, and *TtELF3-*WT defined as the days after sowing (DAS) at which half of the first ear has emerged past the ligule. Upper and lower hinges represent the first and third quartiles (25th and 75th percentiles), the middle hinge represents the median value, whiskers represent the third quartile + 1.5*interquartile range (IQR) and the first quartile – 1.5*IQR, triangle represents the mean value, and black dots represent individual replicates (*n* = 5). (B) Mean *F_m'_* of lines shown in (A) in LL with SEM bars (*n* = 24). (C) relative amplitude error (RAE) where points below the dashed line are considered rhythmic (D) circadian period length (hours) and (E) amplitude for genotypes inplot (B). Box jitter plots as described above, note plotted points in (C-E) correspond to those where data were successfully fitted by the FFT-NLLS model in Biodare2. Significant differences (*P* < 0.05) calculated in R using the Kruskal–Wallis test followed by post-hoc Dunn's test.

Because the allelic variation of *TaELF3* was associated with heading date but not circadian rhythms in the MAGIC parents and RILs, we investigated whether *ELF3* affects heading and is a circadian oscillator component in wheat by examining tetraploid TILLING mutant lines carrying point mutations encoding stop codons in both *TtELF3-A1* and *TtELF3-B1,* or both (Alvarez et al., 2016) (Supplemental Figure 9). The *Ttelf3-*null line headed first (48.7 days) with *TtELF3-*WT heading significantly (*P* < 0.0001) later (56.4 days) (Figure 5A), as previously reported (Alvarez et al., 2016). There was no significant difference (*P* = 0.98) in the heading date between *Ttelf3-*Anull (51.0 days) and *Ttelf3-*Bnull (52.4 days), suggesting that the *TtELF3-A* and *TtELF3-B* homoeologues contribute equally to heading date (Figure 5A). However, they were both significantly later heading than *Ttelf3-*null (*P* < 0.0001) and significantly earlier than *TtELF3-*WT (*P* < 0.0001).

We found that functional *ELF3* is required for robust circadian rhythms in continuous light and that single wild-type *ELF3* homoeologues can maintain circadian function in the absence of other functional homoeologues. There were robust circadian rhythms of *F_m'_* in *TtELF3-*WT (RAE 0.16 ± 0.05), *Ttelf3-*Anull (RAE 0.14 ± 0.04), and *Ttelf3-*Bnull (RAE 0.15 ± 0.07) but no robust circadian rhythms in *Ttelf3-*null (RAE 0.53 ± 0.19) (Figure 5, B and C). The arrhythmic phenotype of *Ttelf3-*null was confirmed using DF and leaf temperature measurements (Supplemental Figure 10, A–F) and was not the result of reduced maximum quantum yield of photosystem 2 (*F_v_/F_m_*) as this was higher for *Ttelf3-*null for the duration of measurement (Supplemental Figure 10, G–I). There was no significant difference in the circadian period of *F_m'_* (*P* > 0.05) between *TtELF3-*WT (24.4 h ± 1.2), *Ttelf3-*Anull (24.3 h ± 0.7), and *Ttelf3-*Bnull (24.9 h ± 1.0). Similarly, there was no significant difference (*P* > 0.05) in the amplitude of *F_m__'_* oscillations between *TtELF3-*WT (241 ± 53), *Ttelf3-*Anull (218 ± 50), and *Ttelf3-*Bnull (236 ± 57).

Our results demonstrate that allelic variation at individual *ELF3* homoeologues is sufficient to alter the heading date without affecting circadian rhythms, indicating that *ELF3* has separable effects on heading date and the circadian clock in wheat.

### Loss of functional *TtELF3* perturbs circadian oscillator gene expression in light/dark cycles and constant light

**Figure 6:**
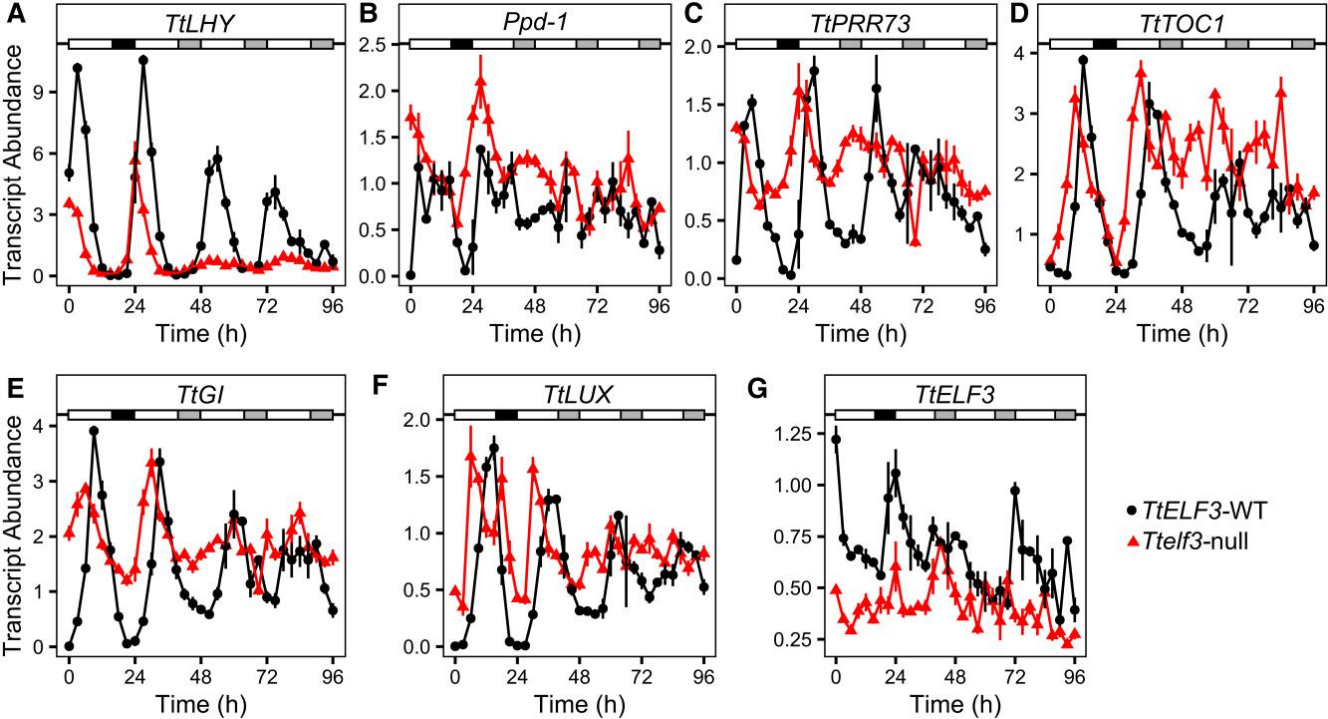
Abundance of wheat circadian clock transcripts in the *TtELF3-WT* and T*telf3-*null lines in light-dark cycles and constant light. (A–G) Mean abundance of circadian oscillator transcripts (*n* = 3–5) in *TtELF3-*WT (circles) and *Ttelf3-*null (triangles) in a 24 h light and dark (LD) cycle in long day conditions (16 h light at 250 µmol m^−2^ s^−1^, 20°C: 8 h dark 16°C, represented by a horizontal grey bar) followed by constant light (LL) and temperature (20°C) from time 24 to 96 h. Transcript abundance (ΔΔCq) is relative to *RP15* and *RPT5A,* (A) *TtLHY,* (B) *Ppd-1,* (C) *TtPRR73,* (D) *TtTOC1,* (E) *TtGI,* (F) *TtLUX,* and (G) *TtELF3*. The white bar represents light, the black bar represents darkness, and the grey bar represents subjective night.

To further investigate the function of *ELF3* in the wheat circadian system, we measured the relative transcript abundance of orthologues of the Arabidopsis circadian oscillator components *LHY, TOC1, Ppd-1* (*PRR37*)*, PRR73, GI, ELF3,* and *LUX* in light-dark (LD) and continuous light (LL) cycles. In LD cycles, all transcripts had rhythmic expression (Figure 6, A–G). Consistent with previous findings in Arabidopsis, *TtLHY* had peak expression at dawn (Figure 6A), the *PRR* orthologues peaked during the photoperiod (Figure 6, B and C) with *TtTOC1* (Figure 6D), *TtGI* (Figure 6E), and *TtLUX* (Figure 6F) peaking around dusk. However, the peak in *TtELF3* abundance is at the end of the night just prior to dawn (Figure 6G), approximately antiphase to the expression of *TtLUX* (Figure 6F). This differs markedly from Arabidopsis in which *ELF3* and *LUX* are co-expressed near dusk (Nusinow et al., 2011; Huang et al., 2016; Ezer et al., 2017). For all transcripts, except *Ppd-1* (Figure 6B), we detected oscillations in relative transcript abundance in constant light, with the phasing in LL consistent with that in LD. These oscillations of circadian transcript abundance are abolished or of very low amplitude in *Ttelf3-*null (Figure 6, A–G). Interestingly, the expression of *Ppd-1* is consistently higher in *Ttelf3-*null in both LD and the first LL cycle (Figure 6B) and the minimum level of the *Ppd-1* homolog *TtPRR73* is also higher in *Ttelf3-*null in LD (Figure 6C) which suggests that ELF3 might act as a negative regulator of *Ppd-1* and *TtPRR73*.

There is a phase advance in peak transcript abundance of approximately 3 h in LD in *Ttelf3-*null compared to *TtELF3-WT* (Figure 6, A–G) except for *TtELF3* transcript abundance where the peak phase is at dawn in both *TtELF3-*WT and *Ttelf3-*null (Figure 6G). The amplitude of transcript abundance under diel conditions is also less in the *Ttelf3-*null line compared to *ELF3*-WT (Figure 6). Together, these data demonstrate that functional *TtELF3* is required for the maintenance of robust oscillations of circadian oscillator gene expression in LL and normal circadian timing in LD.

### *LUX* loss of function disrupts the wheat circadian clock

The antiphase expression of wheat *ELF3* and *LUX* raises the possibility that the EC might not be formed, or functions differently, from that in Arabidopsis. Previous studies in diploid wheat have identified *TmLUX* as a circadian oscillator gene (Gawronski et al., 2014), so we investigated whether *LUX* also contributes to circadian rhythms in hexaploid wheat, and whether the functionality of one homoeologue is sufficient to maintain robust oscillations. We obtained three hexaploid Japanese cultivars, Chogokuwase, Minaminokomugi, and Geurumil that have variation at *TaLUX* associated with the flowering time (Mizuno et al., 2012). Chogokuwase is an extra early flowering variety and is the offspring of Geurumil and Minaminokomugi. Chogokuwase carries predicted non-functional mutations in all three homoeologues of *TaLUX* (Supplemental Figure 11). Chogokuwase also carries a *TaVRN-D1* spring allele and a photoperiod insensitive *Ppd-D1a* allele. Geurumil has the same predicted non-functional alleles of *TaLUX* and *Ppd-D1a* but has a winter growth habit due to lacking the *VRN-D1* spring allele. Minaminokomugi carries the non-functional *Talux-a1* allele, but wild-type alleles of *TaLUX-B1* and *TaLUX-D1,* in addition to carrying the *TaVRN-D1* and *Ppd-D1a* alleles (Mizuno et al., 2016).

**Figure 7:**
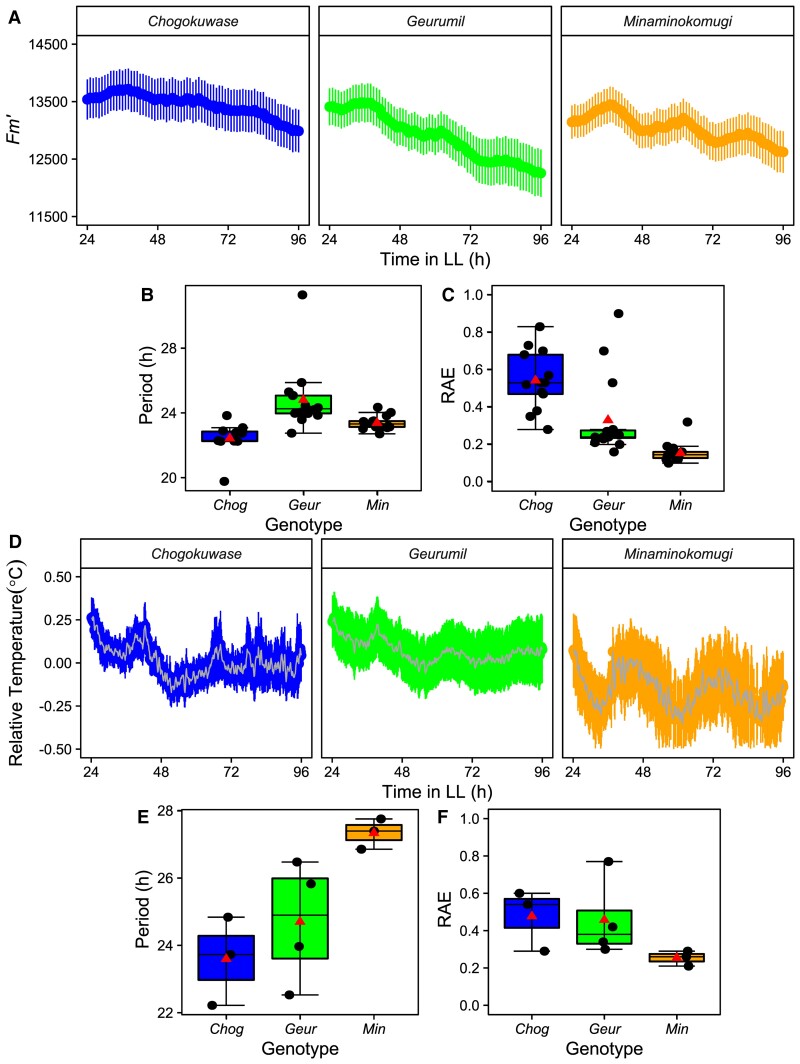
Functional *LUX* is required for the maintenance of robust circadian oscillations under constant light in hexaploid wheat. (A) Mean *F_m'_* for Chogokuwase, Geurumil, and Minaminokomugi in constant light (LL) (*n* = 16). (B) Mean period and (C) relative amplitude error (RAE) for (A) calculated using FFT-NLLS (Biodare2). Upper and lower hinges represent the first and third quartiles (25th and 75th percentiles), the middle hinge represents the median value, whiskers represent the third quartile + 1.5*interquartile range (IQR) and the first quartile – 1.5*IQR, triangle represents the mean value and dots represent individual replicates. (D) Mean Chogokuwase, Geurumil, and Minaminokomugi leaf temperature relative to the background in LL (*n* = 4). (E) mean period and (F) RAE for plot (D). Error bars are SEM, for (D) every 20th point plotted for clarity.

Minaminokomugi maintained robust oscillations of chlorophyll fluorescence (RAE 0.16 ± 0.01) and leaf temperature (RAE 0.26 ± 0.02) (Figure 7, A–C). In contrast, Chogokuwase, which lacks functional copies of *TaLUX,* is arrhythmic for PF (RAE 0.56 ± 0.06) and Geurumil, which is also lacking functional *TaLUX*, has reduced robustness of PF oscillations (RAE 0.33 ± 0.05) compared to Minaminokomugi (Figure 7, A–C). Similarly, there is a decreased robustness of oscillations in leaf surface temperature for Chogokuwase and Geurumil (RAE 0.48 ± 0.09 and RAE 0.46 ± 0.11) which appear to dampen to arrhythmia after the first two true LL cycles (Figure 7, D–F). We conclude that functional *TaLUX* is required for robust circadian rhythms in hexaploid wheat.

## Discussion

### Wheat *ELF3* contributes to heading date and circadian rhythms independently

The eight MAGIC parent varieties provided the opportunity to measure the likely contribution of the *ELF3* homoeologues to heading date and circadian rhythms. Of the MAGIC parents, Brompton, Hereward, Rialto, and Xi-19 have a predicted glycine674 (G|G) substitution variant of *TaELF3-B1* and, in addition, Xi-19 has an inverted copy of *TaELF3-D1,* likely arising from a past introgression event. These variations in *TaELF3* alleles have no discernible impact on circadian rhythms (Figure 4), but are associated with measurable effects on heading date in the field (Supplemental Figure 12).

Consistent with previous studies, *ELF3* is a candidate for *Eps-D1 loci* (Zikhali et al., 2016). We have extended the findings concerning the *Eps-D1* locus by demonstrating that rather than being within a deletion, a copy of *ELF3-D1* is present in Cadenza (as well as Xi-19, Spark, Cordiale, Tonic, and Maris Fundin) but does carry candidate sequence variations. *ELF3-D1* lies within an introgression region that contains an inversion relative to the Chinese Spring D genome (Figure 3) and current data cannot rule out other candidate genes within the introgression and/or inversion region. Based on the analysis of the 820k SNP data, we find that several descendants of the cultivar Tonic carry the putative introgression, as do several other unrelated cultivars and six Watkins lines from India, China, UK, Cyprus, Turkey, and Yugoslavia. Introgression is also likely present within Chinese elite germplasm (Wang et al., 2016). As a result, we hypothesize that this region predates modern breeding efforts. The same genomic region has been identified as being under selection in a collection of bread wheat accessions dating from 1790 to 1930, though in that study, it was presumed to be a deletion rather than introgression (Przewieslik-Allen et al., 2021). While the origin of the introgression is uncertain, the presence of the Jagger/Cadenza *TaELF3-D1* intronic deletion in *Ae. speltoides* and *T. timopheevii* (Supplemental Figure 5) and the clustering outside of *Aegilops tauschii* lines (Supplemental Figures 6 and 7) supports an origin outside of the D genome, more closely related to the G or S genome. The increase in quantity and quality of genomic resources for diverse accessions and wheat-relative species will help in determining the origin of this introgression.

Our data also identify *TaELF3-B1* as a candidate gene underlying the *Eps-B1* locus. We found that a G|G variant in *TaELF3-B1,* which segregates with *Eps-B1,* caused a delay in heading and flowering, compared to the ancestral serine674 variant (A|A) (Supplemental Figure 12), in agreement with the previous observations in Avalon (Zikhali et al., 2016). It was previously concluded that G|G is the ancestral variant (Zikhali et al., 2016) but our evidence suggests that it is more likely that A|A is ancestral because the G|G allele is globally rare, present in 4.87% of lines included in the 1000 Exome dataset, in one of the 62 wheat HapMap panel lines (Supplemental Table 2) and three of the 14 10+ Wheat Genome project lines (Supplemental Table 2), and the ancestral serine residue is highly conserved, present in all sequenced grasses. However, within UK wheat germplasm (as defined by locality on wheat GRIS), the G|G allele is more common and is found in nine of the 13 UK lines we have investigated (Supplemental Table 3). The later flowering G|G allele may be more favored in northern regions (Langer et al., 2014).

We found that the allelic variation at *TaELF3* had no detectable effect on circadian rhythms in the MAGIC parents or early and late heading RILs. Epistasis between *ELF3* and *Ppd-D1* has previously been reported for flowering time (Faure et al., 2012; Alvarez et al., 2016). However, we find that variation at *Ppd-A1* has no effects on circadian rhythms or modification of circadian phenotypes in *Ttelf3*-nulls (Supplemental Figure 13). We also find no evidence of epistasis between *eps-B1* and *eps-D1* in the regulation of circadian rhythms because Xi-19 has both *eps-B1* and *eps-D1,* but was not distinguishable from the rest of the population in terms of circadian behavior.

**Figure 8:**
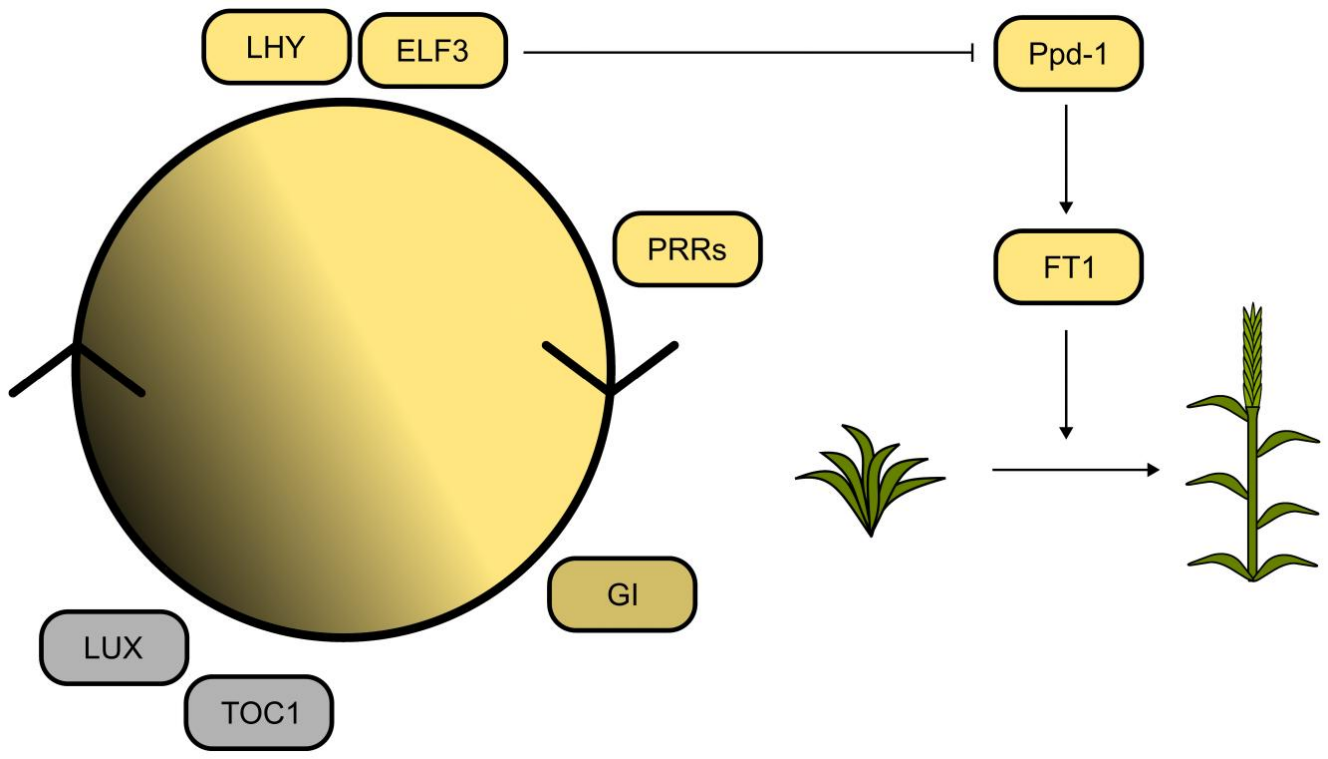
ELF3 functions in the circadian oscillator and regulates the heading of wheat. Genes associated with the circadian oscillator are positioned and shaded relative to the time of day at which their transcript abundance is highest in the diel cycle. The progression of the circadian oscillator is indicated by the circle. Lines indicate potential regulatory links with outputs and other pathways. Pointed arrow head represent activation and blunt arrowhead represents repression.

Using the loss of function lines, we demonstrate that *ELF3* regulates heading and is required for circadian rhythms in wheat. While the complete loss of functional *TtELF3* causes arrhythmia of PF (Figure 5, B–E & Supplemental Figure 10, G–I), leaf temperature (Supplemental Figure 10, D–F), and gene expression in LL (Figure 6), loss of function of either the A or B homoeologue is without effect on circadian rhythms if the other homoeologue is functional (Figure 5, B–E). This contrasts with the effect on heading, where loss of either the A or B homoeologue function results in an advance in heading date, relative to wildtype, and the magnitude of this advance is intermediate relative to the double null mutant (Figure 5A). These results, coupled with the increased expression in *Ttelf3-*null of the floral promoter *Ppd-1* (Figure 6B) and *FT1* (Supplemental Figure 14), and the finding that ELF3 directly binds the *Ppd-1* promoter to repress *Ppd-1* expression (Alvarez et al., 2022), suggest that wheat ELF3 functions as a core circadian oscillator component, while also having a direct effect on flowering, independent of its role in the circadian oscillator (Figure 8).

Loss of functional *TtELF3* advanced the circadian phase in LD. This demonstrates that one role of *ELF3* is to contribute to the correct alignment of the internal phase to the external phase, which is a primary function of circadian oscillators. The earlier phasing of gene expression in LD cycles is similar to barley containing the *eam8.w* allele where the peak phase of *HvCCA1, HvTOC1,* and *HvGI* expression was also earlier (Faure et al., 2012). Similarly, the same study reported the loss of rhythmic oscillations in gene expression upon transition to LL in barley. We found that functional *TtELF3* is required for high amplitude *TtLHY* rhythms and that *TtELF3* loss of function mutants has increased trough transcript abundance of *TtGI* (Figure 6E). The similarity of these observations to those in barley and rice lines carrying *ELF3* mutations (Zhao et al., 2012; Boden et al., 2014) suggests that *ELF3* genes might have similar functions across grasses.

Wheat PHYTOCHROME B (PHYB) and PHYC are required for accelerated heading in long days (Chen et al., 2014; Pearce et al., 2016; Kippes et al., 2020; Bouché et al., 2022) and PHYB and PHYC are required for the induction of *Ppd-1* expression during night-breaks in wheat (Pearce et al., 2017). In Arabidopsis, PHYB co-locates at sites bound by the EC (Ezer et al., 2017), and in Brachypodium, ELF3 appears to affect PHYC activation of *Ppd-1* physical interaction (Gao et al., 2019). Together, these results support the hypothesis that wheat ELF3 may act as an integrator of light signaling downstream of the phytochromes and upstream of Ppd-1 (Kippes et al., 2020) placing it within one of the wheat photoperiod pathways (Shaw et al., 2020) (Figure 8).

### An altered role for the evening complex (EC) in wheat

Binding of the Arabidopsis EC to target promoters requires LUX, as both ELF3 and ELF4 lack DNA binding domains, and it is thought this might explain the similar timing of the transcript accumulation (Nusinow et al., 2011). However, in wheat, the maximal abundance of *ELF3* and *LUX* transcripts is separated by 12 h (Figure 6, F and G). This difference in the peak time of *ELF3* and *LUX* transcript abundance also occurs in *T. monococcum* (Alvarez et al., 2016), rice (Zhao et al., 2012), and between *ELF3-like1* and *LUX* in maize, sorghum, and foxtail millet (Lai et al., 2020). This suggests that the temporal separation of *ELF3* and *LUX* expression might be a general feature of cereal circadian oscillators. Wheat also lacks a clear orthologue of *AtELF4,* and instead, contains *ELF4-like* genes (Calixto et al., 2015). *ELF4-like* genes might have a conserved role because the expression of a barley *ELF4-like* gene restored circadian rhythms to an Arabidopsis *elf4-2* mutant (Kolmos et al., 2009). There is further evidence for the conservation of EC function in monocots because Brachypodium ELF3 binds Arabidopsis ELF4 and LUX, and can complement Arabidopsis *elf3-2* mutants (Huang et al., 2017). The temporal separation of wheat *ELF3* and *LUX* transcript abundance does not preclude the formation of an EC complex, but could suggest one is not formed, or occurs at a different time in the diel cycle to Arabidopsis. Whether through an EC or not, our data demonstrate that functional *ELF3* and *LUX* are required for robust circadian rhythms in wheat (Figures 6–8).

While it is notoriously difficult to attribute plant promoter *cis*-elements to specific patterns of expression (Harmer and Kay 2005), the difference in timing of *TaELF3* and *TaLUX* expression compared to their Arabidopsis orthologues might arise because of promoter sequence differences. Within 5,000 bp upstream of the three *TaELF3* homoeologues transcription start sites, the most common motifs are linked to morning-phased expression (37 morning, 20 evening, and 7 midnight) (Supplemental Table 6). The general composition of the *TaELF3* promoters is similar to *TaLHY* (22 morning, 9 evening, and 3 midnight) (Supplemental Table 7) but different to the evening expressed *TaLUX* promoters where the most abundant motifs are associated with evening phased expression (32) compared to morning (23) and midnight (12) (Supplemental Table 8). In particular*, TaELF3* promoters contain Morning Element 1 (ME1) and Morning Element 2 (ME2) motifs, associated with morning-phased expression (Michael et al., 2008), which are also found in the promoter region of the dawn-expressed *TaLHY* homoeologues (Supplemental Table 7), but are absent in the *AtELF3* promoter (Supplemental Table 6). The *TaELF3* promoters also contain further morning-related motifs such as the multiple Hormone up at dawn 1 (HUD1) and Hormone up at dawn 2 (HUD2) elements (Michael et al., 2008) which are less frequent in the *AtELF3* promoter (Supplemental Table 6).

### Wheat *ELF3* might be a promising breeding target

*ELF3* might be a suitable target for breeding new lines with heading date better adapted to the local environment in the practice of chronoculture (Steed et al., 2021). In other species such as pea, lentil, and barley, allelic variation in *ELF3* has permitted the cultivation of these crops at different latitudes (Weller et al., 2012; Zakhrabekova et al., 2012; Lundqvist 2014). While a complete loss of functional *ELF3* in wheat significantly advances flowering time, severely disrupts circadian rhythms, and decreases the spikelet number (Alvarez et al., 2016), we have demonstrated that altering the function of particular homoeologues of *ELF3* can affect heading without a measurable disruption to the circadian oscillator (Figure 5) and the potential associated yield penalties (Dodd et al., 2005), providing some functional copies of *ELF3* are present. It might be possible by combining different alleles of *ELF3* with alleles of the other circadian clock genes, to precisely tune flowering time suited to the growing environment. This could be tested by measuring the flowering time in selected Cadenza TILLING lines (Krasileva et al., 2017) containing mutations in other circadian clock genes, as Cadenza contains both the *TaELF3-B1* Ser674Gly SNP and the 1D introgression. In addition to the *ELF3* alleles described in this work, we have also identified further *ELF3* alleles including the absence of *ELF3-A1* in Julius (Supplemental Table 9), highlighting a range of *ELF3* alleles within modern wheat cultivars which could be incorporated into breeding programs. The variation at *ELF3* might have occurred because of the positioning of *ELF3* within the subtelomeric region (Aguilar and Prieto 2020) which has enhanced levels of recombination which can lead to an increased number of sequence and structural variants. This has been observed for other regions; recent studies have reported that a deletion at the distal end of chromosome 4AL is associated with altered heat tolerance (Zhai et al., 2021) and a deletion at the distal end of 5AL encompasses the *Reduced height 12* (*Rht12*) locus which is associated with dwarfing (Sun et al., 2019). Second, variation at *ELF3* may have been selected by past breeding efforts, leading to a change in allele frequencies, due to an advantageous adaptation of the flowering time to local climates similar to that seen for the selection of *Ppd-1* alleles based on latitudinal cline (Cockram et al., 2007) and altitude (Guo et al., 2020). A more systematic understanding of circadian clock gene alleles and their combinations will allow for a more targeted approach in optimizing flowering time without impacting the functioning of the circadian clock.

## Materials and methods

### Plant material

The eight MAGIC wheat (*T. aestivum*) parents; Alchemy, Brompton, Claire, Hereward, Rialto, Robigus, Soissons, and Xi-19 were obtained from NIAB (National Institute of Agricultural Botany, UK). The *Ttelf3*-null and *TtELF3-*WT lines were donated by Jorge Dubcovksy (Alvarez et al., 2016) where the *Ttelf3-*null line is the progeny of bulked *elf3-null/Ppd-A1b* and the *TtELF3-*WT line is the progeny of bulked *ELF3WT/Ppd-A1b* (Table 4). By outcrossing, we selected lines that contained just the *Ttelf3-A1* mutation (herein *Ttelf3-*Anull) or just the *Ttelf3-B1* mutation (*Ttelf3-*Bnull). The Chogokuwase, Minaminokomugi, and Geurumil lines were donated by Hidetaka Nishida (Mizuno et al., 2012).

**Table 4:** Wheat lines used in this study. GRU refers to the germplasm resource unit at the John Innes Centre, Norwich

Species	Cultivar	Genotype	Reference
***Ae. speltoides***			GRU 2140023
***T. aestivum***	Alchemy		(Mackay et al., 2014)
***T. aestivum***	Brompton		(Mackay et al., 2014)
***T. aestivum***	Claire		(Mackay et al., 2014)
***T. aestivum***	Hereward		(Mackay et al., 2014)
***T. aestivum***	Rialto		(Mackay et al., 2014)
***T. aestivum***	Robigus		(Mackay et al., 2014)
***T. aestivum***	Soissons		(Mackay et al., 2014)
***T. aestivum***	Xi-19		(Mackay et al., 2014)
***T. aestivum***	Cadenza		GRU WBCDB0013-PG1
***T. aestivum***	Jagger		GRU PANG0006
***T. aestivum***	Spark		GRU WBCDB0052
***T. aestivum***	Chinese Spring		
***T. aestivum***	Chogokuwase		(Mizuno et al., 2016)
***T. aestivum***	Minaminokomugi		(Mizuno et al., 2016)
***T. aestivum***	Geurumil		(Mizuno et al., 2016)
***T. aestivum***	Axona		GRU W3580
***T. aestivum***	Cordiale		GRU W10003
***T. aestivum***	Maris Fundin		GRU W1357
***T. aestivum***	Tonic		GRU W3572
***T. monococcum***			GRU T1040057
***T. sphaerococcum***			GRU 1210007
***T. timopheevii***			GRU 116002
***T. turgidum***	Kronos	*Ttelf3-*null line	(Alvarez et al., 2016)
***T. turgidum***	Kronos	*TtELF3-*WT	(Alvarez et al., 2016)
***T. turgidum***	Kronos	*Ttelf3-*Anull	In house
***T. turgidum***	Kronos	*Ttelf3-*Bnull	In house

### Plant growth conditions

Seeds were surface-sterilized by washing once in 70% (v/v) ethanol for one minute (Fischer Scientific, UK), once in autoclaved water for one minute, followed by 15 min in 10% (v/v) sodium hypochloride solution (Fischer Scientific, UK) and then washed a further three times in autoclaved deionized water. Seeds were sown directly onto 96 well modular trays (P G Horticulture Ltd, UK), containing a 1:1 mix of Levington M2 potting compost (Levington, UK) and vermiculite (fine, William Sinclair Horticulture, UK), and treated with the insecticide Intercept 70W (0.02 g L^−1^ soil, Bayer, Germany). Plants were grown in growth chambers (Conviron, Canada) under long days and constant temperature (16 h L: 8 h D; 200 µmol m^−2^ s^−1^; 20°C or 16 h L: 8 h D; 400 µmol m^−2^ s^−1^; 22°C) or in growth cabinets (see specific methods).

### Field trials

In 2012/2013 a fully replicated yield trial of 784 F_5_ MAGIC lines at the NIAB experimental farm in Cambridge, UK was scored by NIAB for the timing of growth stages on Zadok's scale (Zadoks et al., 1974; Mackay et al., 2014). Each plot was assessed every two days for its growth stage based on representative plants at the center of each plot. Once a plot reached GS55 (half of the ear emerged above the flag leaf ligule), the date was logged. The phenotype was assessed with growth stage measurements scored as days after sowing. In the 2013/2014 field season, the same growth stage phenotypes were collected from a fully replicated yield trial of 784 F_6_ MAGIC lines.

### Analysis of field phenotypes

Asreml-R 3.0 (Gilmour et al., 1997) was used to minimize or remove spatial effects in phenotype data due to field variation.

Marker genotypes and their respective chromosomal groupings from (Gardner et al., 2016) were used. The genetic map was reordered visually using the R package mpMapInteractive (Shah 2013) to improve ordering and consider physical ordering information from IWGSCRefSeqv1. Recombination fractions were recalculated in mpMap (Huang and George, 2011) for each chromosome using mpestrf(). Recalculating recombination fractions on a per chromosome basis with the existing marker groupings substantially reduced computation resources required and enabled the use of finer recombination bins (r < − c(0:5/600, 2:20/200, 11:50/100) compared to the default values. Genetic map lengths were recalculated using the Kosambi mapping function in computemap().

To reduce the computational effort, a skimmed genetic map with only unique mapping locations was used to calculate the probability that each location on a genome was inherited from each parent and conduct QTL mapping in MPWGAIM only (Verbyla et al., 2014). A marker for each unique coordinate was chosen, which had the lowest missing calls and preferably, a clear BLAST hit to the IWGSCRefSeqv1 reference sequence, which was used to assign physical mapping coordinates. Out of 18,089 mapped markers 4,458 were chosen for founder probability calculations.

To test and validate our pipeline and analysis tools, we used them to interrogate a data set describing the flowering time in a 2011/2012 nursery of the NIAB eight-Parent MAGIC population that had previously been analyzed using Bayesian Networks (Scutari et al., 2014). Reanalysis of these data using MPWGAIM (Verbyla et al., 2014) detected 13 significant QTLs for flowering time with significant founder effects (Supplemental Table 1).

This analysis identified a major QTL (18.3% genetic variation explained, LOGP 18.33) between the markers Kukri_c27309_590 (48.57 cM) and BS00064538_51 (56.64 cM), defining a maximum mapping interval of 31.81–34.23 Mb between the two mapping bins as several markers map to the 56.64 cM bin. The mean founder effects of the QTL predict a significant acceleration of flowering time for Soissons, which is the only parent carrying the photoperiod insensitive *Ppd-D1a* allele (Bentley et al., 2013) and, furthermore, *Ppd-D1* maps within the QTL (33.95 Mb). This analysis of the effect of *Ppd-D1a* on flowering time validated MPWGAIM and our QTL mapping pipeline in MAGIC.

### Synteny analysis between Chinese Spring and Jagger 1d

A conserved haplotype block between Cadenza and Jagger at the distal end of chromosome 1D was identified using the Crop Haplotypes web browser [Crop Haplotypes (crop-haplotypes.com)] (Brinton et al., 2020). Physical locations of genes on the Chinese Spring 1D chromosome (IWGSCRefSeqv1.1) and Jagger 1D chromosome (PGSBv2.0) were mapped using the mapviewer v2.6.1 (https://10wheatgenomes.plantinformatics.io/mapview) and then confirmed manually using EnsemblPlants.

### SNP mining and analysis

820k and 35k SNP array data were obtained from the CerealsDB website (Wilkinson et al., 2012) and the 1000Exome data from the 1,000 Exomes project website (http://wheatgenomics.plantpath.ksu.edu/1000EC/). Mapping of 35k and 820k SNPs to the IWGSCv1.1 Ref sequence was obtained from EnsemblPlantsv51. SNPs for the Cadenza/Jagger haplotype (chr1D: 482.3Mb – end of chromosome 1D) were extracted and heterozygous calls were set as missing. Distance matrices and phylogenetic trees were constructed using the R package “ape” (Paradis and Schliep 2019); 100 bootstrap permutations were run also using the R package “ape”.

To investigate the prevalence of Ser674Gly SNP in a broad range of germplasm, we analyzed variation data from multiple sources including the 820k SNP genotyping array (Winfield et al., 2016), the wheat HapMap panel (Jordan et al., 2015), the 1,000 wheat Exome project (He et al., 2019), and the 10+ Wheat Genome project (Walkowiak et al., 2020). We found that the Ser674Gly SNP was also present in the HapMap panel as chr1B_685645813 and within the 1000 Exome data as scaffold48561_337271.

### Phylogenetic tree construction

*ELF3-D1* orthologous groups were retrieved from public repositories. Coding sequences were aligned using the MCoffee aligner on default settings (Wallace et al., 2006). Maximum Likelihood (ML) Phylogenetic trees were built using CLC Genomics Workbench 21.0.3. The best substitution model was determined using the Model testing function before running Molecular Phylogenetic analysis by the Maximum Likelihood method with 100 permutations for Bootstrap values. The *ELF3* gene from *Brachypodium distachyon* was used as an outgroup.

### Reverse-transcription quantitative PCR

The *TtELF3*-WT and *Ttelf3*-null lines were sown and grown in two antiphased LED growth chambers (Conviron) under long day conditions (16 h L: 8 h D; 250 µmol m^−2^ s^−1^, 20°C day: 16°C night). Sampling commenced 16 days post-sowing, the first true leaf was sampled every 3 h for 96 h. After the first 24 h of sampling, the cabinet was switched to constant light and temperature (250 µmol m^−2^ s^−1^, 20°C). Total RNA was extracted from leaf samples using the KingFisher Flex purification system (Thermofisher scientific) in conjunction with the Maxwell® HT simplyRNA Kit (Promega). RNA concentration was determined using the Nanodrop ND-1000 (Thermofisher scientific). DNA contamination was removed using the Arcticzyme HL-DNAse kit. RNA integrity was checked using the Fragment AnalyzerTM (AATI, USA) before cDNA was synthesized from extracted RNA using the High-Capacity cDNA reverse-transcription kit (Applied Biosystems). Three technical replicates of gene-specific products were amplified in 10 µl reactions using Power SYBR Green Master mix on a CFX384 Touch Real-Time PCR detection system (Bio Rad). Gene expression levels were determined relative to the expression of two housekeeping genes, *RP15* and *RPT5A,* that were selected using the GeNorm algorithm (Vandesompele et al., 2002) (Table 5).

**Table 5:** Primers used in this study

Gene	Sequence 5′-3′
***TtCCA1***	F: CCTGGAATTGGAGATGGAGA R: TGAGCATGGCTTCTGATTTG
***TtELF3***	F: TCTCCAGATGATGTTGTCGGT R: CTCGAACACTTGGACAGCAAA
***TtGI*** ***TtFT1***	F: GGTAGGTGATAGACGGCACTT R: GTGCTACAGATGGGATGCTTG F: TGAGGACCTTCTACACACTCG R: ACCGGGGATATCTGTCACAAG
***TtLUX***	F: ACAAGCGGTTCGTGGAGG R: CCTGCATCCGCTTGACGTA
***TtPpd-1***	F: CCTGTGGACTGTCGATCTCAA R: CAAGGGATGGCAGCGATAATG
***TtPRR73***	F: TCCCGAAGTTCCTCTCTTTCC R: AGCGGTAGTGGCAATGACA
***TtTOC1***	F: GGCATGGCACTTCATTCAGTT R: GCACATTCATACCAGCAGGAC
***RP15***	F: GCACACGTGCTTTGCAGATAAG R: GCCCTCAAGCTCAACCATAACT
***RPT5A*** ***TaELF3-D1***	F: GCTGGCTCGTTCAACTGATG R: GGACCAAGCGTTCTGATTACTC F: CAACGGCGCTTCTAATCTG R: AAACCCGCTGCTGACTGTATA

For quantification of *TaELF3* transcript abundance, Cadenza, Chinese Spring, Claire, Paragon, and Robigus seed was sown and grown in a Conviron growth room (16 h L: 8 h D; 400 µmol m^−2^ s^−1^, continuous 22°C. After 14 days, leaf samples were taken and RNA extracted using the RNAeasy plant mini kit (Qiagen, UK) with an on-column DNAse digest (Qiagen, UK). RNA concentration was determined on the Nanodrop ND-1000 and cDNA was synthesized from 500 ng RNA using the RevertAid First Strand cDNA synthesis kit (Thermo Scientific, UK). Technical duplicates of gene-specific products were synthesized in 10 µl reactions using the Rotor-Gene SYBR Green PCR kit (Qiagen, UK) on a Rotor-Gene 6000 Real-Time PCR machine (Qiagen, UK).

### PCR of *ELF3-D1*

DNA was extracted using the DNeasy plant mini extraction kit (Qiagen, UK). The presence of *ELF3-D1* was confirmed by PCR using the *TaELF3-D1* primers listed in Table 5. PCR was performed using the DreamTaq Green PCR kit (Thermo Scientific, UK) on a ProFlex PCR system (Thermo Scientific, UK). PCR reaction conditions were as follows: 3 min at 95°C; 40 cycles of 30 s at 95°C, 30 s at 68°C, 90 s at 72°C; 15 min at 72°C. PCR products were sanger sequenced (Azenta Life Sciences, UK) with sequences mapped to *ELF3-D1* (Supplemental Table 10) using the Geneious 11.1.1 Read Mapper tool with default settings.

### Delayed chlorophyll fluorescence and prompt chlorophyll *a* fluorescence imaging

Leaf samples were taken from the upper half of the first true leaf of 16-day-old seedlings (non-vernalized) grown and entrained in Conviron growth cabinets and were cut into 3 mm by 3 mm pieces and placed on solid 0.8% (w/v) bactoagar, 0.5 x MS Vitamin medium (M3900, Sigma-Aldrich, UK) supplemented with 0.5 µM benzyl-aminopurine, poured in black 96-well imaging plates (Greiner, UK). Delayed fluorescence (Gould et al., 2009) was measured for 60 s in an LB985 Nightshade (Berthold, UK) camera. Between measurements of DF light was supplied from blue (470 nm) red (660 nm) and LED arrays at 100 µmol m^−2^ s^−1^ during the experiment. The temperature was maintained at 20°C.

Chlorophyll *a* fluorescence and calculations of parameters were carried out using a CFimager (Technologica Ltd, Colchester, UK) and the image processing scripts provided by the manufacturer. Chlorophyll fluorescence images were captured using a Stingray F145B ASG camera (Allied Vision Technologies, UK) through an RG665 long pass filter to exclude blue light from the LEDs. When imaging under continuous light, leaf fragments were exposed to 40 min of 100 µmol m^−2^ s^−1^ of blue light followed by a saturating pulse of 6,172 µmol m^−2^ s^−1^ blue light for 800 ms, then 20 min of darkness (with non-actinic measuring light on) followed by a second saturating pulse of 6,172 µmol m^−2^ s^−1^ for 800 ms.

Circadian measures of amplitude, phase, period, and relative amplitude error were calculated using the FFT-NLLS method in Biodare2 (www.biodare2.ed.ac.uk) (Zielinski et al., 2014), where necessary data were normalized to between −1 and 1 using the normalization feature of Biodare2.

### Heading date measurements in *TtELF3* kronos lines

Seeds were sown as described above and moved to long day conditions (Conviron growth cabinet, 16 h light: 8 h dark; 400 µmol m^−2^ s^−1^, 22°C). After two weeks, seedlings were transplanted to 10 cm pots. Heading (GS55) was scored once per day.

### Direct contact leaf temperature measurements

Leaf surface temperature relative to background temperature was measured using an in-house built device and provided an alternative measure of circadian rhythms caused by circadian-regulated changes in the stomatal aperture (Dakhiya and Green, 2019). The temperature logger was validated by measuring circadian rhythms of “in-phase” and “anti-phase” wheat leaves where peak temperature, and therefore maximum stomatal closure, occurred during the subjective night (Supplemental Figure 15). Leaf temperature measurements were taken from fully expanded flag leaves of 4-week-old plants under continuous light and temperature (200 µmol m^−2^ s^−1^, 20°C) that were previously grown under long days (16 h L: 8 h D; 200 µmol m^−2^ s^−1^, 20°C day: 15°C night) in a growth cabinet (Conviron, UK).

Wheat was grown in 10 cm pots. Circadian measurements of leaf temperature were taken when the plants were four weeks old. Type-K thermocouples were attached to the leaf such that the end of the thermocouple was pressed onto the surface of the fully expanded flag leaf. This was kept in position by positioning a paperclip onto the leaf and fixing the paperclip to a stake. The thermocouples were connected to an Arduino Mega using the Adafruit 1778, a 10-bit D-A converter, and a x20 amplifier giving a resolution of ± 0.1°C with a DS3231 RTC added to keep time; the temperature was logged every 30 s. The background temperature was measured using unattached thermocouples. Leaf temperatures were averaged using a 60-point moving average; the background temperature was then subtracted from leaf temperature to give leaf temperature relative to the background.

### Statistical analysis

All statistical analysis was performed in R (Version 4.0.5, R foundation for statistical computing, Vienna, Austria) using default parameters. Statistical differences between circadian phenotypes and the MAGIC parents (Figure 4), and between *TaELF3-B1* promoter haplotype and *TaELF3* expression (Supplemental Figure 2) were analyzed using ANOVA with post-hoc Tukey HSD, associated *P* values reported in the text. To analyze the effect of the *TtELF3* genotype on GS55 and circadian phenotypes (Figure 5), Kruskal–Wallis test followed by post-hoc Dunn's test was used with associated *P* values reported in the text. To analyze the relationship between heading phenotype and circadian period (Supplemental Figure 8) correlation analysis was performed with associated *P* values reported in the text.

## Accession numbers

All sequences used in this study are publicly available. The 10+ wheat genome and UK cultivar scaffold sequences were obtained through BLAST search using resources linked via https://10wheatgenomes.com/data-repository/using the Chinese Spring gene sequences as query. The IWGSC RefSeq Annotation v1.1 gene identifiers used for BLAST queries can be found in Supplemental Table 10. All sequences are also available in Ensembl Plants under “cultivars” http://plants.ensembl.org/Triticum_aestivum/Info/.

## Supplemental Data

The following materials are available in the online version of this article.

**Supplemental Figure S1**. ELF3 Ser674 is highly conserved in monocots.

**Supplemental Figure S2**. Relative expression of *TaELF3* in five wheat cultivars that differ in *TaELF3-B1* promoter (C)_n_ polymorphic *loci*.

**Supplemental Figure S3**. The Cadenza/Jagger *TaELF3-D1* intronic deletion is present within Cordiale, Maris Fundin, and Tonic.

**Supplemental Figure S4**. Simplified pedigree diagram shows the relationship between different wheat varieties that carry the Jagger/Cadenza *TaELF3-D1* allele.

**Supplemental Figure S5**. The Cadenza/Jagger *TaELF3-D1* intronic deletion is present within *Ae. speltoides* and *T. timopheevii*.

**Supplemental Figure S6**. Phylogeny of the 1D Jagger/ Cadenza shared haplotype using the 820k SNP dataset from CerealsDB.

**Supplemental Figure S7**. Phylogeny of *ELF3-D1* gene.

**Supplemental Figure S8**. Circadian rhythms of chlorophyll *a* fluorescence in early and late heading MAGIC RILs are not associated with genotype.

**Supplemental Figure S9**. The location of mutations within the A and B sub-genome copies of *TtELF3*.

**Supplemental Figure S10**. *Ttelf3-null* is arrhythmic for DF and relative leaf temperature in continuous light which is not a result of leaf stress.

**Supplemental Figure S11**. The location of mutations in the different sub-genome copies of *LUX*.

**Supplemental Figure S12**. The Ser674Gly single-nucleotide polymorphism (SNP) is a candidate for the causal *Eps-B1* SNP.

**Supplemental Figure S13**. There is no significant difference between the circadian rhythms of Kronos and Ttelf3-null lines containing the *Ppd-A1a* and *Ppd-A1b* alleles.

**Supplemental Figure S14**. Relative *FT1* transcript abundance in *Ttelf3-null* and *TtELF3-WT* in light-dark and continuous light conditions.

**Supplemental Figure S15**. In-phase and anti-phase leaf temperature rhythms in continuous light.

**Supplemental Table S1**. GS55 2012 QTLs.

**Supplemental Table S2**. The chr1B_685645813/ Ser674Gly SNP is globally rare.

**Supplemental Table S3**. The Ser674Gly SNP segregates with heading date in UK varieties.

**Supplemental Table S4**. Haplotype analysis of the *TaELF3-D1* region between exon 2 and exon 3 (see Fig 3).

**Supplemental Table S5**. Alignment of 300 kb surrounding *ELF3-D1* in 10+ wheat genome lines.

**Supplemental Table S6**. Promoter motif analysis of *TtELF3.*

**Supplemental Table S7**. Promoter motif analysis of *TtLHY.*

**Supplemental Table S8**. Promoter motif analysis of *TtLUX.*

**Supplemental Table S9**. Total number of SNPs within the coding sequence of *ELF3* in the 10+ Wheat Genomes project cultivars.

**Supplemental Table S10**. IWGSC RefSeq Annotation v1.1 gene identifiers used for BLAST queries.

## Supplementary Material

kiac544_Supplementary_Data
