# Supplementary material for: Wheat EARLY FLOWERING 3 affects heading date without disrupting circadian oscillations
Source: Plant Physiol. 2022 Dec 1;191(2):1383–403. doi: 10.1093/plphys/kiac544 (PMC9922389; doi:10.1093/plphys/kiac544)
Supplement: kiac544_Supplementary_Data [file kiac544_supplementary_data.pdf]

Supplemental Material

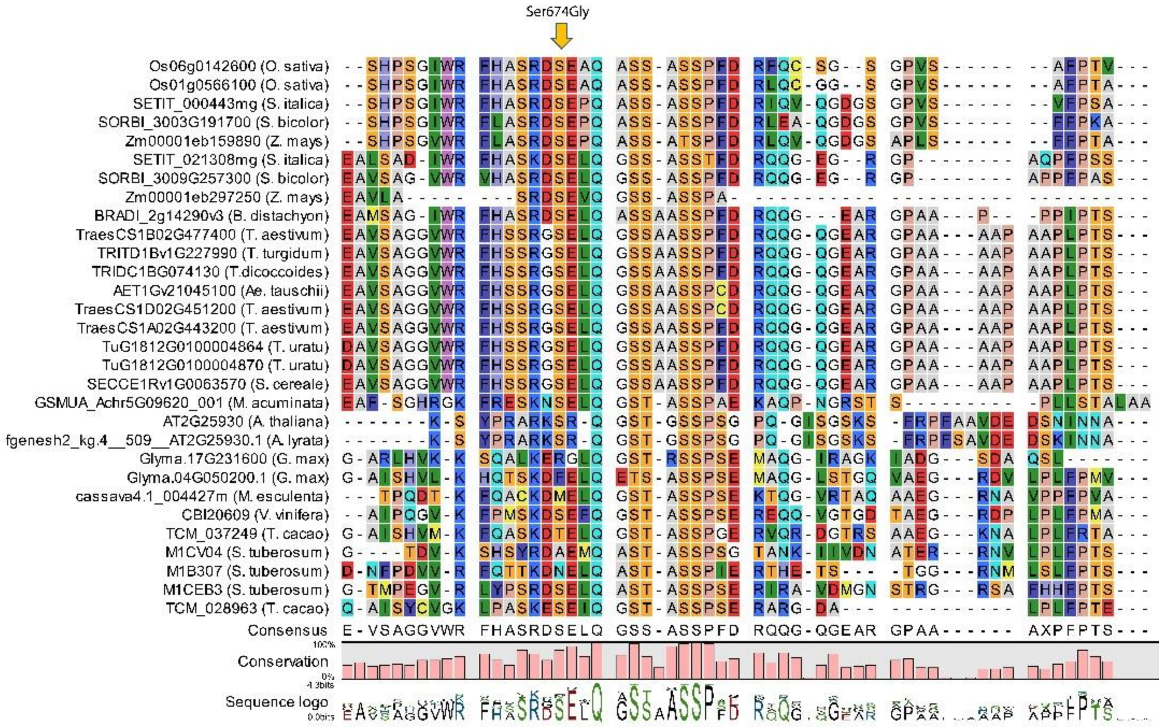

Supplemental Figure S1. ELF3 Ser674 is highly conserved in monocots. MCoffee alignment visualised in CLC Genomics Workbench, amino acids coloured based on RasMol colour scheme.

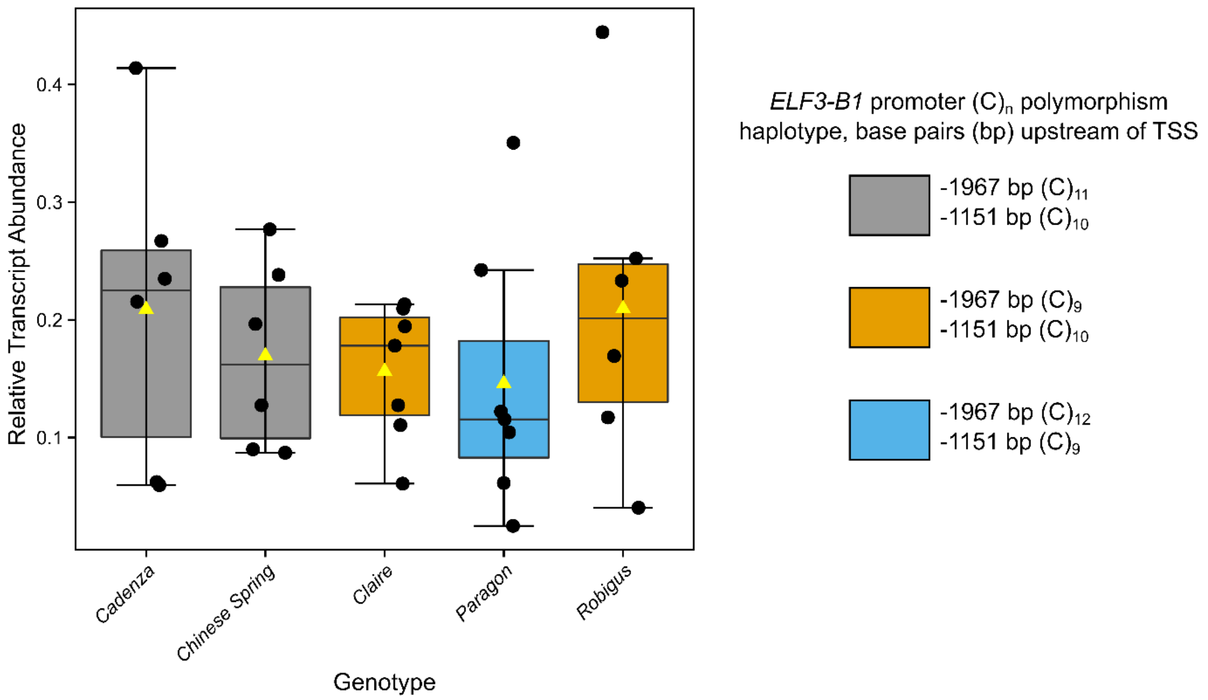

Supplemental Figure S2. Relative expression of *TaELF3* in five wheat cultivars that differ in *TaELF3-B1* promoter $(C)_n$ polymorphic *loci.* Expression of *TaELF3* relative to *RP15* and *RPT5A* at dawn. Upper and lower hinges represent the first and third quartiles (25th and 75th percentiles), the middle hinge represents the median value, whiskers represent third quartile + 1.5*interquartile range (IQR) and first quartile – 1.5*IQR, yellow triangle represents mean value and black dots represent individual replicates.

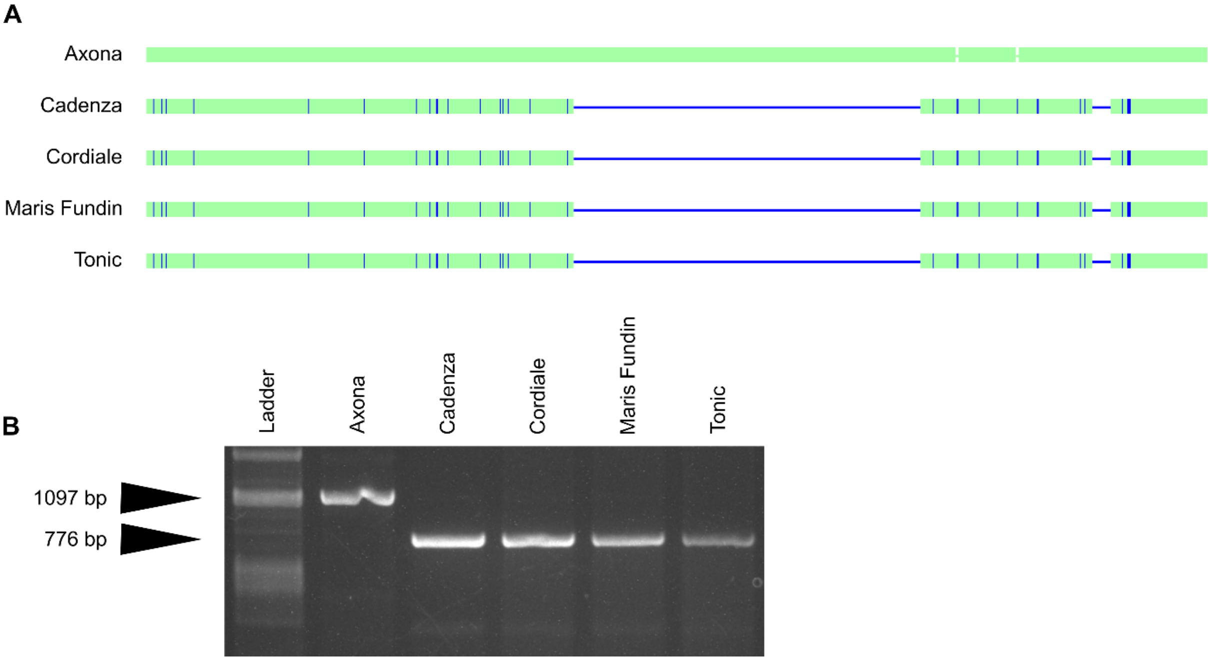

Supplemental Figure S3. The Cadenza/Jagger *TaELF3-D1* intronic deletion is present within Cordiale, Maris Fundin and Tonic.
(A) Sequence alignment for PCR fragments between exon 2 and 3 of *TaELF3-D1* (see Fig 3) and (B) Agarose gel separation of PCR products. A PCR product at 776 bp is indicative of the presence of the introgressed and inverted region.

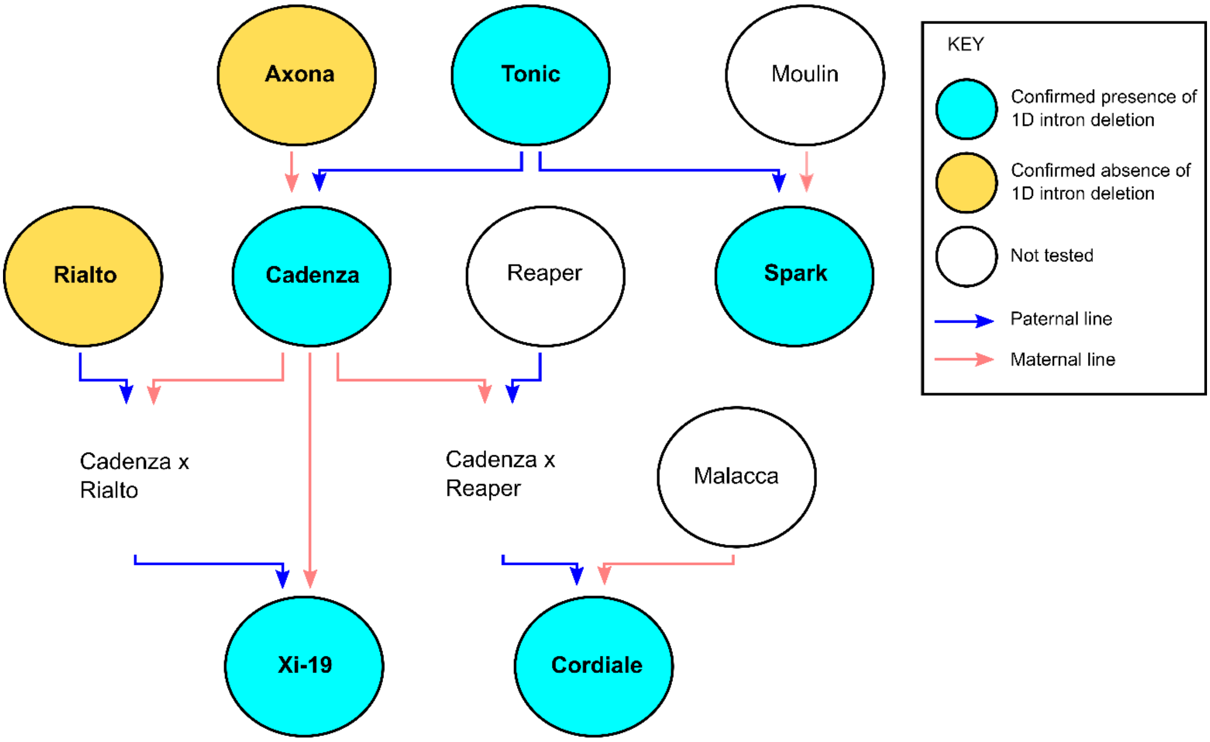

Supplemental Figure S4. Simplified pedigree diagram shows the relationship between different wheat varieties that carry the Jagger/Cadenza *TaELF3-D1* allele.
Pedigree diagram constructed from relationships described at https://wheatpedigree.net and (Fradgley et al., 2019). Note Cadenza x Rialto and Cadenza x Reaper are not available as germplasm for testing.

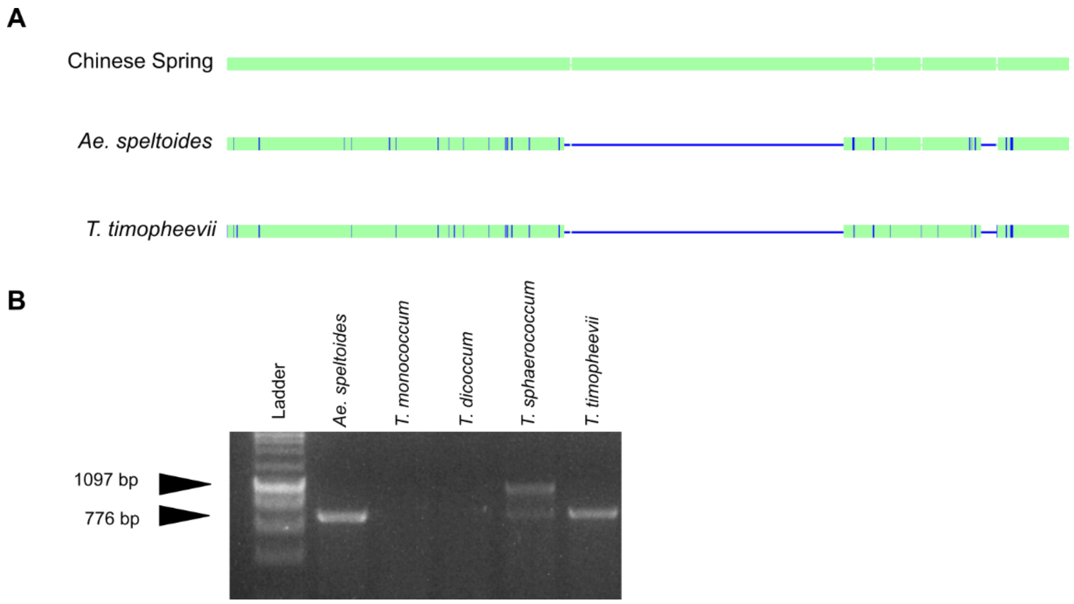

Supplemental Figure S5. The Cadenza/Jagger *TaELF3-D1* intronic deletion is present within *Ae. speltoides* and *T. timopheevii.*

(A) Sequence alignment for PCR fragments between exon 2 and 3 of *ELF3-D1* (see Fig 3) and (B) Agarose gel separation of PCR products. A PCR product at 776 bp is indicative of the presence of the introgressed and inverted region.

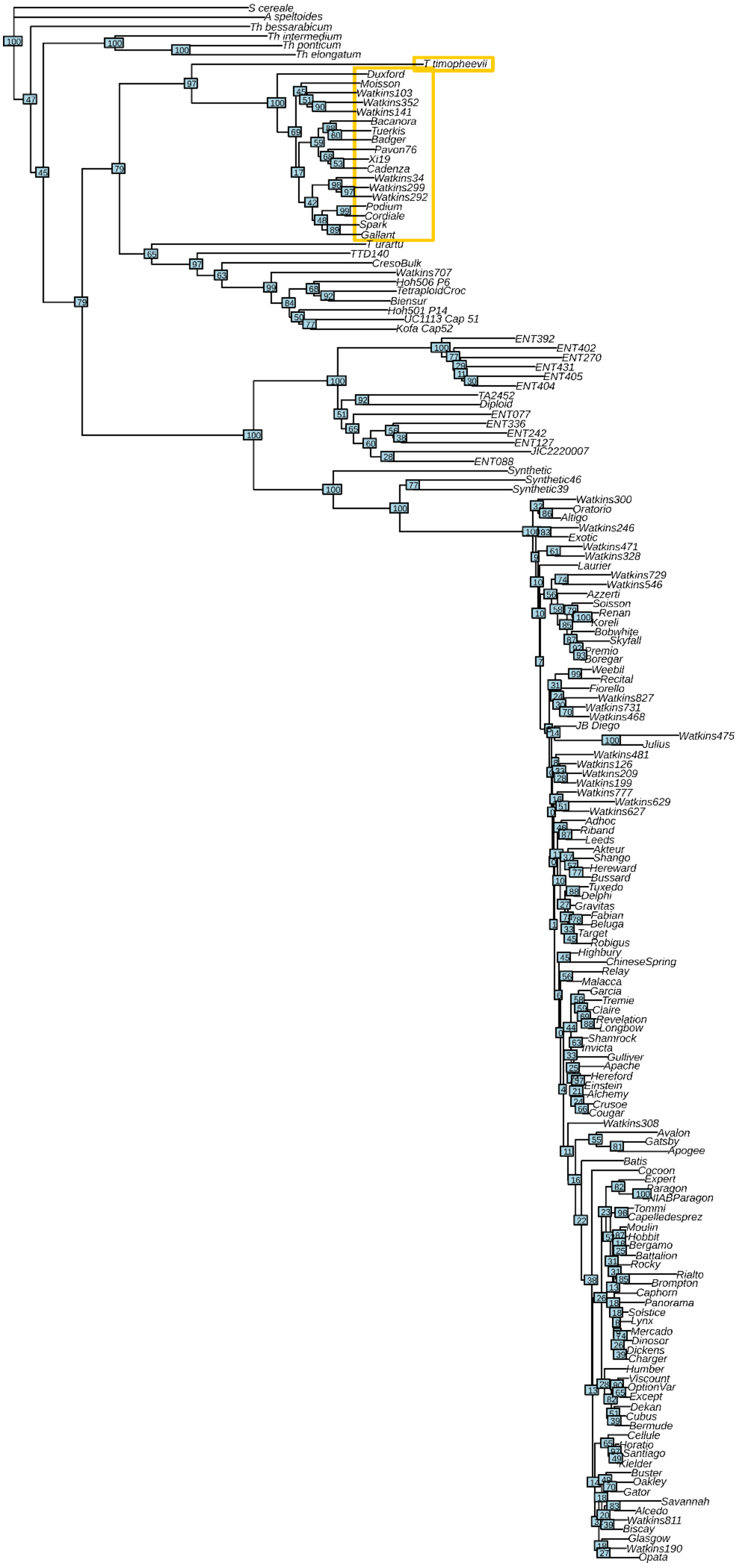

Supplemental Figure S6. Phylogeny of the 1D Jagger/ Cadenza shared haplotype using the 820k SNP dataset from CerealsDB.

Neighbour Joining Tree and bootstraps created from 820k SNPs using R package “ape”. All SNPs mapping to the CS coordinates (1D:482.83Mb until 1D end) of the shared Cadenza and Jagger haplotype were used. Heterozygotes were set as missing. Yellow boxes highlight varieties associated with the 1D introgression.

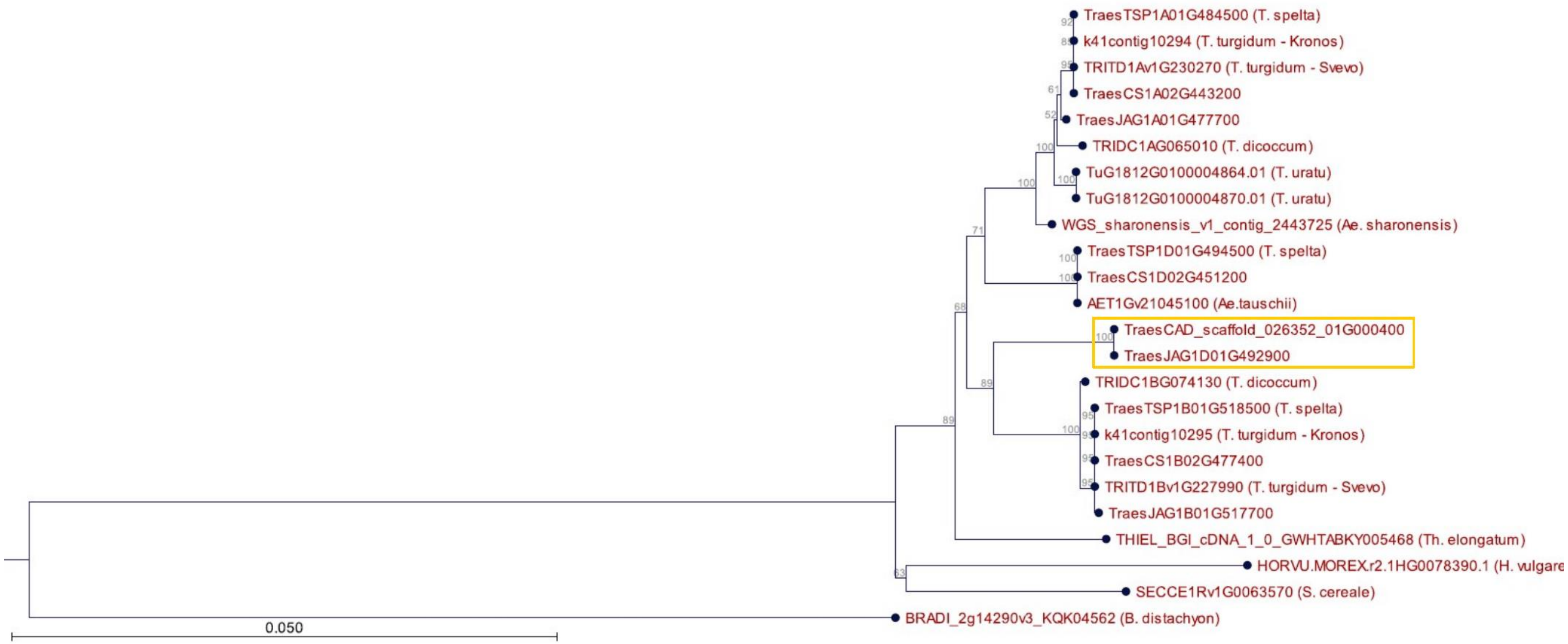

Supplemental Figure S7. Phylogeny of *ELF3-D1* gene.
Maximum Likelihood phylogeny based on T-coffee alignment of publicly available coding sequences. Cadenza and Jagger highlighted by yellow box.

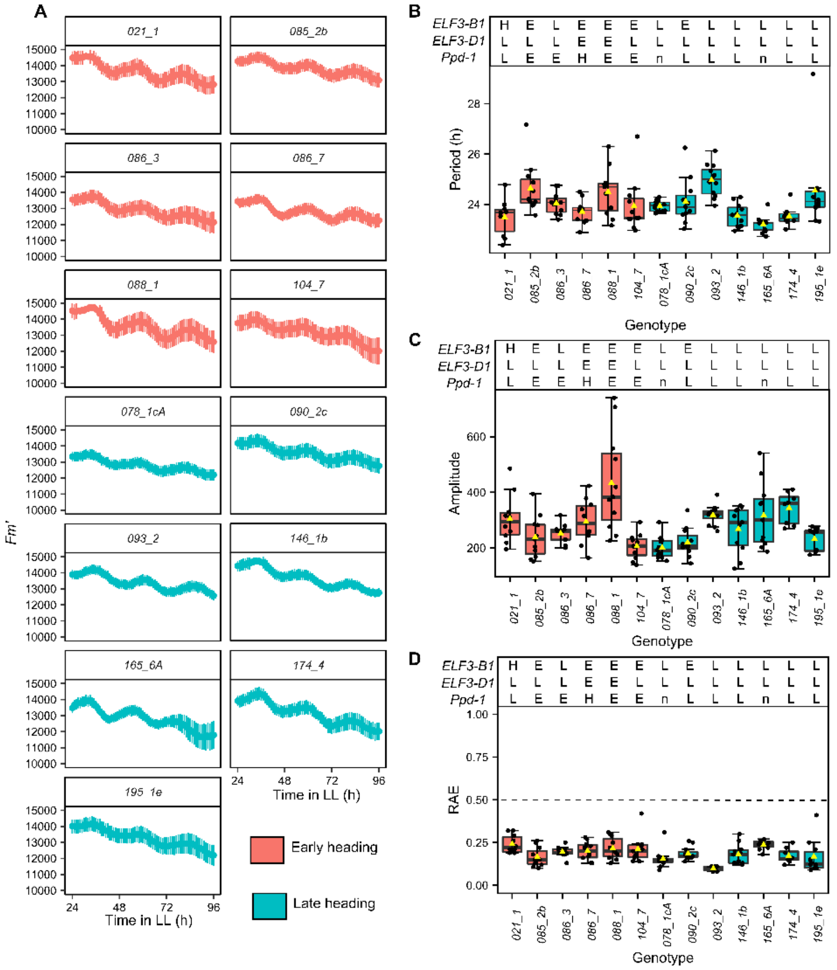

Supplemental Figure S8. Circadian rhythms of chlorophyll *a* fluorescence in early and late heading MAGIC RILs are not associated with genotype.
(A) Mean *Fm'* in continuous light (LL) for a selection of early heading (red) and late heading (cyan) MAGIC RILs from 2014 with error bars representing SEM (n = 12). (B) circadian period (C) circadian amplitude and (D) relative amplitude error (RAE). (B-C) calculated using FFT-NLLS analysis (Biodare2), upper and lower hinges represent the first and third quartiles (25th and 75th percentiles), the middle hinge represents the median value, whiskers represent third quartile + 1.5*interquartile range (IQR) and first quartile – 1.5*IQR, yellow triangle represents mean value and black dots represent individual replicates. Top of (B-D) represents the alleles of *ELF3-B1, ELF3*-D1 and *Ppd*-1 where E = early heading associated allele, L = late heading associated allele, H = heterozygous and n = no sequencing information. Horizontal dashed line in (E) represents rhythmic cut-off where RAE < 0.5 is considered rhythmic.

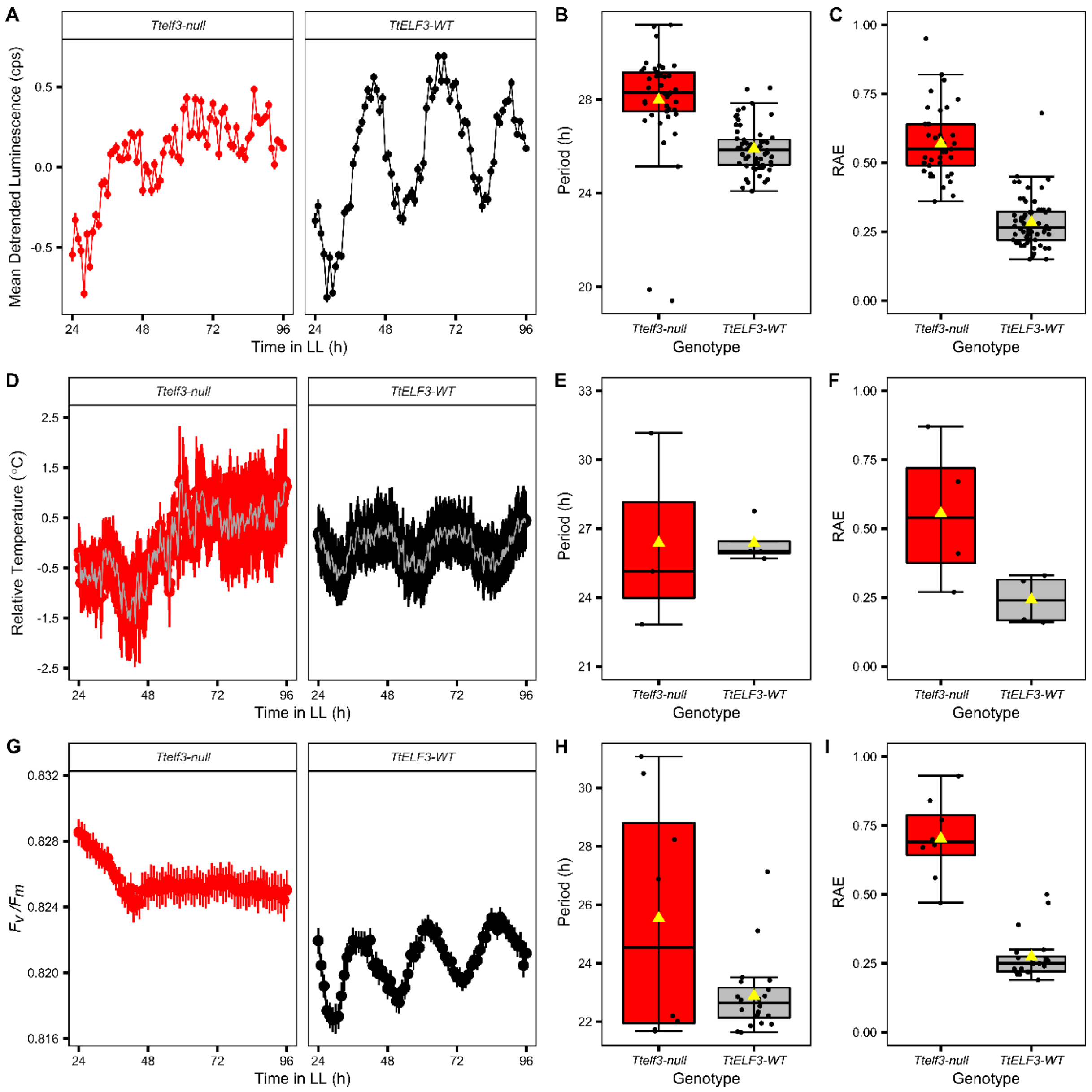

Supplemental Figure S10. *Ttelf3-null* is arrhythmic for DF and relative leaf temperature in continuous light which is not a result of leaf stress.
(A) Mean delayed chlorophyll fluorescence (DF) luminescence in counts per second (cps) normalised to -1 to 1 using Biodare2 in continuous light (LL) with error bars representing SEM (n = *TtELF3*-WT 63, *Ttelf3*-null *72*). (B) Amplitude and (C) relative amplitude error (RAE) for *TtELF3*-WT (black) and *Ttelf3*-null (red) in (A) calculated using FFT-NLLS analysis in Biodare2 where upper and lower hinges represent the first and third quartiles (25th and 75th percentiles), the middle hinge represents the median value, whiskers represent third quartile + 1.5*interquartile range (IQR) and first quartile – 1.5*IQR, yellow triangle represents mean value and black dots represent individual replicates. (D) mean leaf surface temperature relative to background and normalised to the mean in LL and constant environmental temperature (20°C) *TtELF3*-WT (black) and *Ttelf3*-null (red) with SEM bars (n = 7), for clarity every 20th point plotted. (E) Circadian period and (F) RAE for plot (D), presented and calculated as for (B-C). (G) Mean F_v/F_m of *TtELF3*-WT (black) and *Ttelf3*-null (red) in LL (n = 24.) (H) Circadian period (hours) and (C) relative amplitude error (RAE) values for plot (G), calculated as for (B-C).

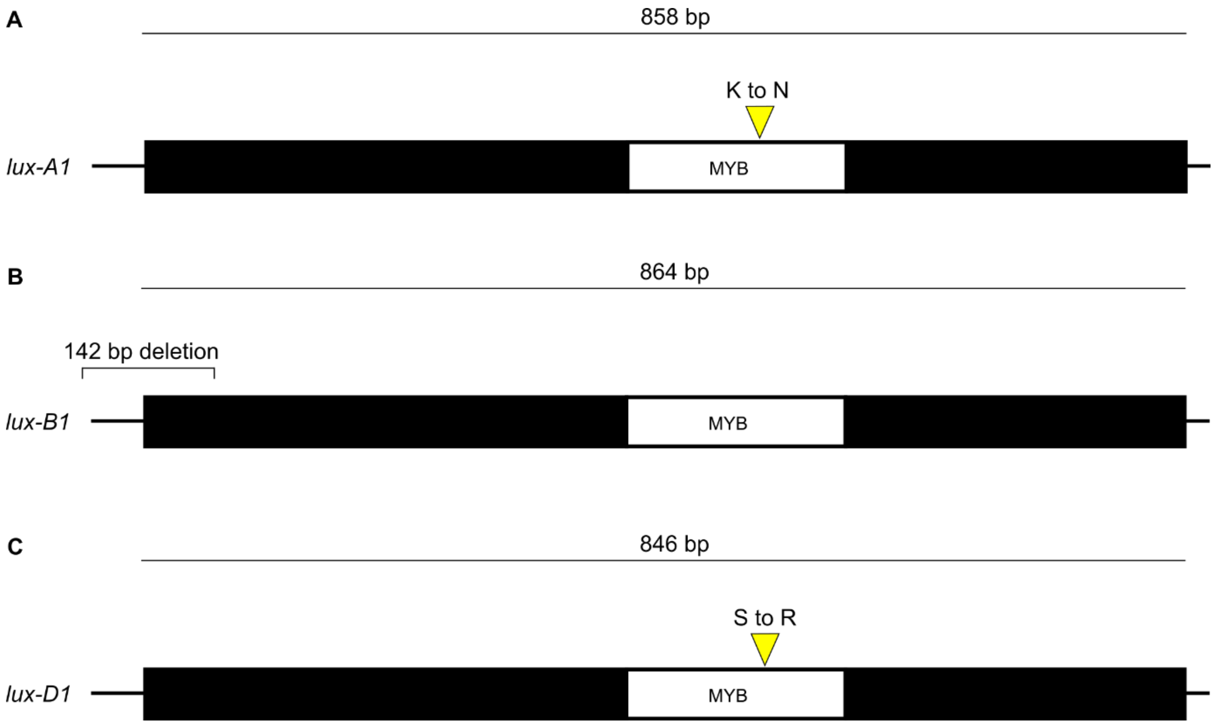

Supplemental Figure S11. The location of mutations in the different sub-genome copies of *LUX.*
(A-C) Diagram of the A sub-genome (A), B sub-genome (B) and D sub-genome (C) copies of *LUX* where black rectangle represents the *LUX* exon drawn 5' to 3' (left-to-right) and the embedded white rectangle denotes the highly conserved MYB-domain. Yellow triangles denote the location of amino acid substitution within the SHAQKYF motif (A, C). (B) line and annotation show the location of 142 bp deletion in the *lux-B1* allele (Mizuno *et al*. 2016)

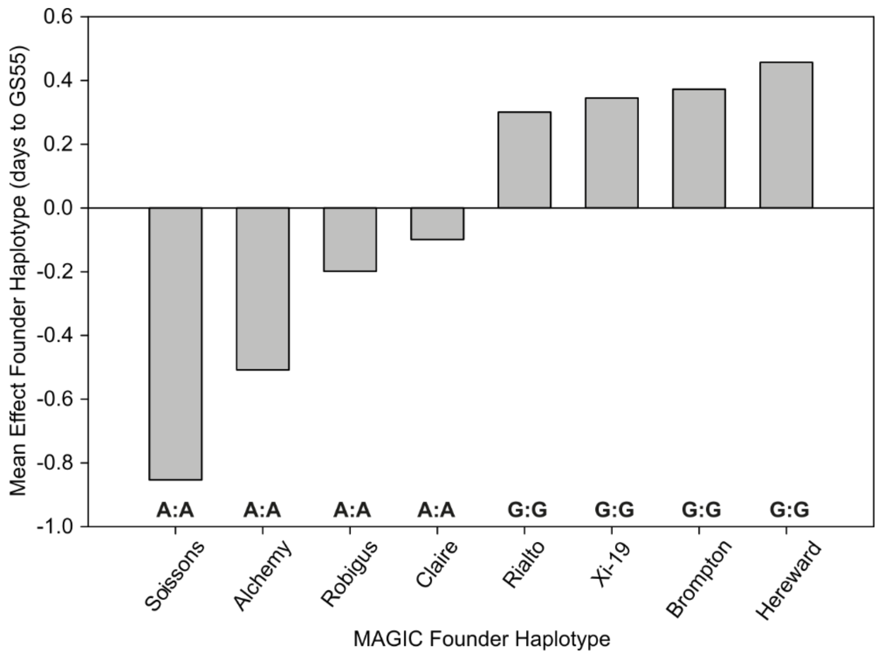

Supplemental Figure S12. The Ser674Gly single nucleotide polymorphism (SNP) is a candidate for the causal *Eps-B1* SNP.

Kompetitive allele specific PCR (KASP) genotype association with MAGIC founder haplotype effects at the *Eps-B1* locus. Founder haplotype effects are calculated as the mean of the 2013 and 2014 founder effect. Genotype of A|A corresponds to homozygous for adenine, encoding the ancestral Serine, whilst G|G corresponds to homozygous guanine encoding the non-ancestral Glycine residue.

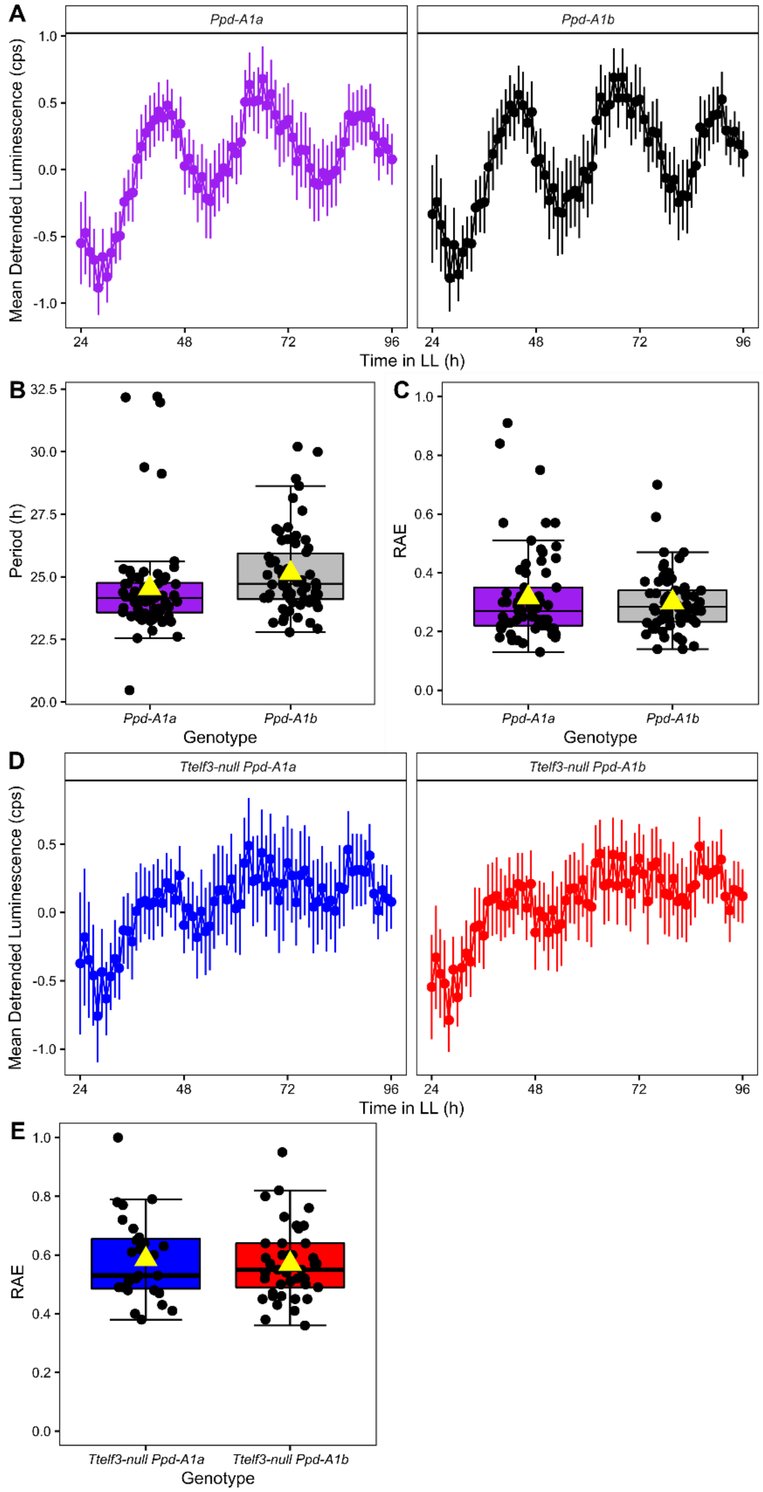

Supplemental Figure S13. There is no significant difference between the circadian rhythms of Kronos and *Ttelf3-null* lines containing the *Ppd-A1a* and *Ppd-A1b* alleles.

(A) Mean delayed chlorophyll *a* fluorescence (DF) luminescence in counts per second (cps) normalised to -1 to 1 using Biodare2 in continuous light (LL) with error bars representing standard deviation (n = *Ppd-A1a* 68, *Ppd-A1b* 62). (B) Amplitude and (C) relative amplitude error (RAE) for *Ppd-A1a* (purple) and *Ppd-A1b* (black) in (A). (D) Mean DF luminescence in counts per second (cps) normalised to -1 to 1 using Biodare2 with error bars representing standard deviation (n = *Ttelf3-null Ppd-A1a* 66, *Ttelf3-null Ppd-A1b* 22) and (E) RAE for *Ttelf3-null Ppd-A1a* (blue) and *Ttelf3-null Ppd-A1b* (red). Amplitude and RAE calculated using FFT-NLLS analysis in Biodare2 where upper and lower hinges represent the first and third quartiles (25th and 75th percentiles), the middle hinge represents the median value, whiskers represent third quartile + 1.5*interquartile range (IQR) and first quartile – 1.5*IQR, yellow triangle represents mean value and black dots represent individual replicates for plants where estimation was possible.

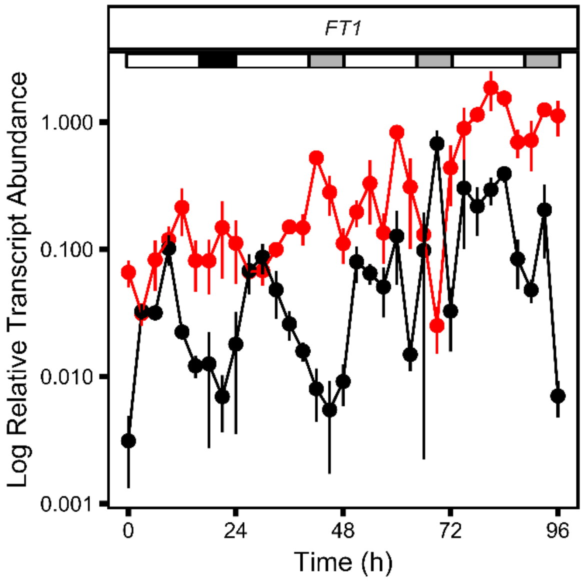

Supplemental Figure S14. Relative *FT1* transcript abundance in *Ttelf3*-null and *TtELF3*-WT in light-dark and continuous light conditions.

Mean *FT1* transcript abundance with SEM bars (n = 3-5) relative to *TtRP15* and *TtRPT5A* expression in *TtELF3*-WT (black) and *Ttelf3*-null (red) in a 24 h light-dark (LD) cycle in long day conditions (16 h light at 250 µmol m^{-2} s^{-1}, 20°C: 8 h dark 16°C) followed by constant light and temperature (20°C) from time 24 to 96 h. Horizontal white rectangle represents light, black rectangle represents darkness and grey rectangle represents subjective night.

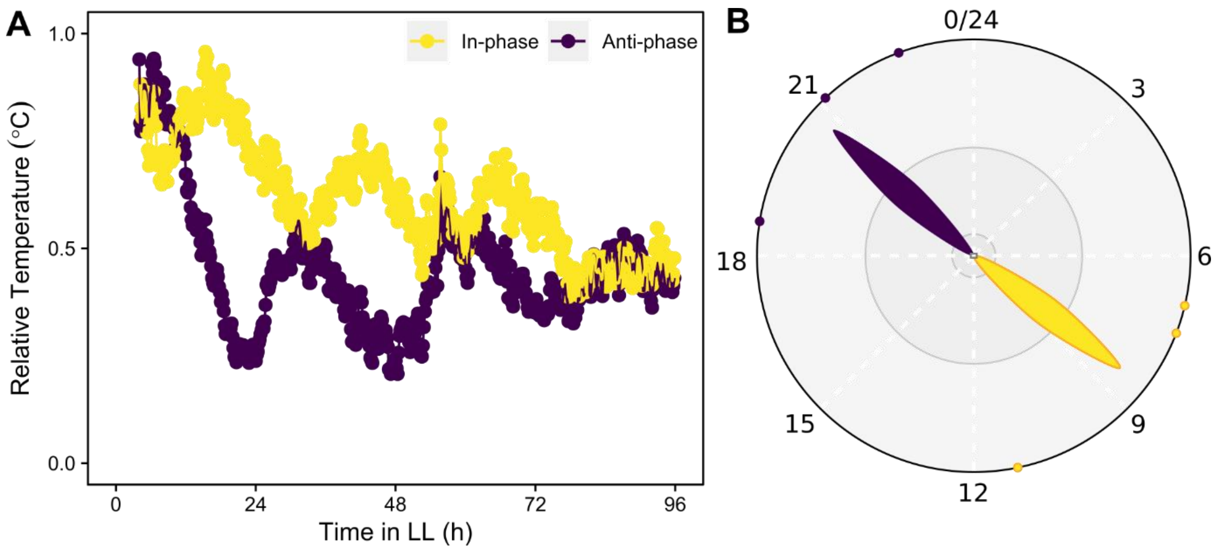

Supplemental Figure S15. In-phase and anti-phase leaf temperature rhythms in continuous light.
(A) Mean leaf temperature relative to background (n = 3) measured in continuous light (LL) of four-week-old cadenza plants entrained to 16 h light:8 h dark cycles in-phase (yellow) or 16 h light: 8 h dark cycles that were 12 hours anti-phased (purple). (B) Phase plot of data shown in (A).

Supplemental Table S1. GS55 2012 QTLs

Output from the reanalysis of the NIAB 2012 GS55 data using MPWGAIM, originally analysed using Bayesian Networks (Scutari et al., 2014). Dist (cM) refers to the markers genetic mapping distance in centiMorgan. Founder corresponds to founder variety in alphabetical order (1 Alchemy, 2 Brompton, 3 Claire, 4 Hereward, 5 Rialto, 6 Robigus, 7 Soissons and 8 Xi-19). Size: Size of effect of each founder allele (days to GS55). LOGP is –log10(p) and corresponds to the overall significance of the QTL as a measure of its strength. Prob: probability QTL is from all founders shown (p). Founder LOGP and Founder Prob are LOGP and Prob for individual founders. % var is the percentage of genetic variance explained by each QTL.

Chr	Left Marker	dist (cM)	Right Marker	dist (cM)	Founder	Size	Founder Prob	Founder LOGP	Prob	% var	LOGP
**1B**	IAAV5516	297.84	BS00001128_51	298.34	1	-0.207	0.399	0.4	0.048	1.6	**1.32**
					2	0.946	0.077	1.11			
					3	0.112	0.445	0.35			
					4	0.318	0.304	0.52			
					5	0.698	0.148	0.83			
					6	-1.119	0.07	1.15			
					7	-1.185	0.021	1.67			
					8	0.437	0.259	0.59			
**1D**	Kukri_c2464_592	18.04	D_contig57829_235	18.21	1	-0.81	0.113	0.95	0.003	2.5	**2.59**
					2	-0.569	0.238	0.62			
					3	1.282	0.042	1.37			
					4	-1.024	0.066	1.18			
					5	0.631	0.27	0.57			
					6	0.849	0.12	0.92			
					7	-1.18	0.036	1.45			
					8	0.82	0.21	0.68			
**1D**	CAP11_c1157_168	100.79	Kukri_c29687_369	100.95	1	0.552	0.254	0.6	0.275	0.9	**0.56**
					2	0.105	0.446	0.35			
					3	-0.11	0.447	0.35			
					4	0.021	0.49	0.31			
					5	-0.044	0.477	0.32			
					6	0.003	0.498	0.3			
					7	0.734	0.134	0.87			
					8	-1.261	0.015	1.82			
**2A**	RAC875_c20273_823	35.38	BS00094817_51	35.88	1	0.45	0.218	0.66	0.035	1.6	**1.45**
					2	0.771	0.163	0.79			
					3	0.133	0.418	0.38			
					4	-1.126	0.032	1.5			

Chr	Left Marker	dist (cM)	Right Marker	dist (cM)	Founder	Size	Founder Prob	Founder LOGP	Prob	% var	LOGP
					5	-0.193	0.404	0.39			
					6	-1.262	0.019	1.71			
					7	0.739	0.11	0.96			
					8	0.487	0.241	0.62			
**2B**	BS00060618_51	317.61	BS00000012_51	318.61	1	0.176	0.417	0.38	0.049	1.6	**1.31**
					2	0.748	0.175	0.76			
					3	1.643	0.022	1.66			
					4	-0.88	0.107	0.97			
					5	-0.565	0.206	0.69			
					6	-0.392	0.267	0.57			
					7	-0.326	0.304	0.52			
					8	-0.404	0.265	0.58			
**2D**	Kukri_c27309_590	48.57	BS00064538_51	56.64	1	0.669	0.293	0.53	0	18.5	**18.33**
					2	2.727	0.009	2.03			
					3	0.215	0.44	0.36			
					4	1.112	0.211	0.68			
					5	0.759	0.276	0.56			
					6	0.897	0.245	0.61			
					7	-6.194	0	7.11			
					8	-0.185	0.437	0.36			
**3A**	wsnp_Ex_rep_c106152_90334299	78.8	Kukri_c51247_322	83.81	1	0.671	0.184	0.74	0.001	3	**2.84**
					2	-1.839	0.006	2.25			
					3	-0.671	0.187	0.73			
					4	0.716	0.155	0.81			
					5	-0.885	0.102	0.99			
					6	0.215	0.374	0.43			
					7	0.247	0.364	0.44			
					8	1.546	0.01	2.01			
**3D**	RAC875_c48773_253	31.11	RFL_Contig2471_119	31.28	1	-0.133	0.442	0.35	0.417	0.7	**0.38**
					2	0.159	0.431	0.37			
					3	-0.351	0.351	0.45			
					4	-0.525	0.276	0.56			
					5	-0.025	0.489	0.31			

Chr	Left Marker	dist (cM)	Right Marker	dist (cM)	Founder	Size	Founder Prob	Founder LOGP	Prob	% var	LOGP
					6	-0.443	0.244	0.61			
					7	1.191	0.021	1.68			
					8	0.128	0.423	0.37			
**4A**	BS00064140_51	133.18	BS00101512_51	134.18	1	0.521	0.273	0.56	0.002	2.4	**2.75**
					2	0.176	0.415	0.38			
					3	0.277	0.376	0.42			
					4	0.266	0.36	0.44			
					5	0.118	0.447	0.35			
					6	0.019	0.488	0.31			
					7	0.727	0.187	0.73			
					8	-2.104	0	3.63			
**5B**	BobWhite_c22572_782	196.48	Kukri_c836_513	196.65	1	-3.398	0.001	3.11	0	6	**3.85**
					2	0.725	0.232	0.63			
					3	0.886	0.158	0.8			
					4	1.621	0.088	1.05			
					5	-0.001	0.5	0.3			
					6	-1.505	0.037	1.43			
					7	0.704	0.206	0.69			
					8	0.968	0.141	0.85			
**6B**	wsnp_Ex_c12450_19850827	215.75	tplb0045b09_1555	216.42	1	0.089	0.461	0.34	0.269	1.1	**0.57**
					2	0.723	0.117	0.93			
					3	-0.552	0.264	0.58			
					4	0.368	0.333	0.48			
					5	-0.111	0.447	0.35			
					6	0.247	0.388	0.41			
					7	0.467	0.299	0.53			
					8	-1.232	0.02	1.71			
**6D**	BS00094895_51	124.37	GENE_4168_925	125.04	1	-0.044	0.472	0.33	0.169	1	**0.77**
					2	-0.093	0.449	0.35			
					3	0.85	0.082	1.09			
					4	-0.622	0.216	0.66			
					5	-0.674	0.198	0.7			
					6	0.592	0.169	0.77			

Chr	Left Marker	dist (cM)	Right Marker	dist (cM)	Founder	Size	Founder Prob	Founder LOGP	Prob	% var	LOGP
					7	-0.56	0.152	0.82			
					8	0.551	0.187	0.73			
**7B**	BS00036788_51	58.06	CAP7_c10566_170	58.22	1	-0.73	0.18	0.74	0.008	2.5	**2.09**
					2	-0.247	0.402	0.4			
					3	0.422	0.312	0.51			
					4	1.264	0.029	1.53			
					5	0.22	0.411	0.39			
					6	1.249	0.065	1.19			
					7	-0.704	0.173	0.76			
					8	-1.473	0.012	1.91			

Supplemental Table S2. The chr1B_685645813/ Ser674Gly SNP is globally rare. Wheat HapMap data of a diverse germplasm collection and the 10+ Wheat genome project (italics). UK varieties highlighted in bold. Genotype of A|A corresponds to homozygous for adenine at the Ser674Gly SNP encoding the ancestral serine whilst G|G corresponds to homozygous guanine encoding the non-ancestral glycine (red).

**Variety**	**Genotype (forward strand)**	**Variety**	**Genotype (forward strand)**	**Variety**	**Genotype (forward strand)**
93	A|A	*Jagger*	*A|A*	PI278297	A|A
102	A|G	*Julius*	*A|A*	PI349512	A|A
403	A|A	klein_chamaco	A|A	PI366716	A|A
407-IV_60	A|A	*Lancer*	*A|A*	PI366905	A|A
AC_Barrie	A|A	M6	A|A	PI382150	A|A
acc1	A|A	*Mace*	*A|A*	PI406517	A|A
acc2	A|A	Marquis	A|A	PI445736	A|A
acc3	A|G	Neepawa	A|A	PI470817	A|A
acc4	A|G	*Norin61*	*A|A*	PI477870	A|A
acc5	A|A	Opata	A|A	PI481718	A|A
Alabasskaja	A|A	***Paragon***	**G|G**	PI481923	A|A
Arina	*G|G*	pavon	A|A	PI565213	A|A
**Avalon**	**A|A**	pbw343	A|A	PI82469	A|A
Cadenza	***G|G***	PI153785	A|A	PI8813	A|A
CDC Landmark	*A|A*	PI166180	A|A	PR267	A|A
CDC Stanley	*A|A*	PI166333	A|A	rac875	A|A
chakwat86	A|A	PI177943	A|A	**Rialto**	**G|G**
cham6	A|A	PI185715	A|A	***Robigus***	***A|A***
Chinese Spring	A|A	*PI190962*	*A|A*	*Sy Mattis*	*A|A*
Claire	***A|A***	PI192001	A|A	**Taxi**	**A|A**
clear_white	A|A	PI192569	A|A	Truman	A|A
dharwar_dry	A|A	PI222669	A|A	Utmost	A|A
Estacao	A|A	PI245368	A|A	vorobey	A|A
hidhab	A|A	PI262611	A|A	*Weebil*	*A|A*

Supplemental Table S3. The Ser674Gly SNP segregates with heading date in UK varieties. Additional genotyping and heading information from Zikhali *et al*., 2016 (Zikhali et al., 2016) TGAC reference sequences (Clavijo et al., 2017). MAGIC founder varieties highlighted in bold, non-ancestral glycine in red.

Variety	Allele	Heading
**Alchemy**	A|A	early
**Claire**	A|A	early
**Robigus**	A|A	early
Avalon	A|A	early
**Brompton**	G|G	late
**Hereward**	G|G	late
**Rialto**	G|G	late
**Xi-19**	G|G	late
Paragon	G|G	NA
Cadenza	G|G	late
Spark	G|G	NA
Badger	G|G	NA
Savannah	G|G	NA

Supplemental Table S4. Haplotype analysis of the *TaELF3-D1* region between exon 2 and exon 3 (see Fig 3). Variant location refers to position relative to Chinese spring *TaELF3-D1* transcription start site, first letter refers to base in Chinese Spring, second letter refers to changed base.

	ELF3-D1 region	Exon 2	Intron 2																									
	**Variant location**	T1832C	C1933T	C1982T	A2028G	C2040A	A2046G	T2056G	A2084G	A2102G	G2104A	G2109C	C2128T	A2161G	2167-2471	T2483C	2504-2505	A2521G	2555	T2562C	2609	2613-2614	2616-2622	2625-2627	2633	2636-2637	G2646C	2651-2653
**Cultivar**	**Haplotype**	**Var 1**	**Var 2**	**Var 3**	**Var 4**	**Var 5**	**Var 6**	**Var 7**	**Var 8**	**Var 9**	**Var 10**	**Var 11**	**Var 12**	**Var 13**	**Var 14**	**Var 15**	**Var 16**	**Var 17**	**Var 18**	**Var 19**	**Var 20**	**Var 21**	**Var 22**	**Var 23**	**Var 24**	**Var 25**	**Var 26**	**Var 27**
Alchemy	H1	T	C	C	A	C	A	T	A	A	G	G	C	A	Ins	T	-	A	-	T	G	GT	Ins	Ins	A	CT	G	CAA
ArinaLrFor	H1	T	C	C	A	C	A	T	A	A	G	G	C	A	Ins	T	-	A	-	T	G	GT	Ins	Ins	A	CT	G	CAA
Brompton	H1	T	C	C	A	C	A	T	A	A	G	G	C	A	Ins	T	-	A	-	T	G	GT	Ins	Ins	A	CT	G	CAA
Cadenza	H2	C	T	T	G	A	G	G	G	G	A	C	T	G	Del	C	AG	G	T	C	-	-	Del	Del	-	-	C	TGG
Chinese Spring	H1	T	C	C	A	C	A	T	A	A	G	G	C	A	Ins	T	-	A	-	T	G	GT	Ins	Ins	A	CT	G	CAA
Claire	H1	T	C	C	A	C	A	T	A	A	G	G	C	A	Ins	T	-	A	-	T	G	GT	Ins	Ins	A	CT	G	CAA
Hereward	H1	T	C	C	A	C	A	T	A	A	G	G	C	A	Ins	T	-	A	-	T	G	GT	Ins	Ins	A	CT	G	CAA
Jagger	H2	C	T	T	G	A	G	G	G	G	A	C	T	G	Del	C	AG	G	T	C	-	-	Del	Del	-	-	C	TGG
Julius	H1	T	C	C	A	C	A	T	A	A	G	G	C	A	Ins	T	-	A	-	T	G	GT	Ins	Ins	A	CT	G	CAA
Landmark	H1	T	C	C	A	C	A	T	A	A	G	G	C	A	Ins	T	-	A	-	T	G	GT	Ins	Ins	A	CT	G	CAA
Mace	H1	T	C	C	A	C	A	T	A	A	G	G	C	A	Ins	T	-	A	-	T	G	GT	Ins	Ins	A	CT	G	CAA
Norin61	H1	T	C	C	A	C	A	T	A	A	G	G	C	A	Ins	T	-	A	-	T	G	GT	Ins	Ins	A	CT	G	CAA
Paragon	H1	T	C	C	A	C	A	T	A	A	G	G	C	A	Ins	T	-	A	-	T	G	GT	Ins	Ins	A	CT	G	CAA
Rialto	H1	T	C	C	A	C	A	T	A	A	G	G	C	A	Ins	T	-	A	-	T	G	GT	Ins	Ins	A	CT	G	CAA
Robigus	H1	T	C	C	A	C	A	T	A	A	G	G	C	A	Ins	T	-	A	-	T	G	GT	Ins	Ins	A	CT	G	CAA
Soissons	H1	T	C	C	A	C	A	T	A	A	G	G	C	A	Ins	T	-	A	-	T	G	GT	Ins	Ins	A	CT	G	CAA
Spark	H2	C	T	T	G	A	G	G	G	G	A	C	T	G	Del	C	AG	G	T	C	-	-	Del	Del	-	-	C	TGG
Stanley	H1	T	C	C	A	C	A	T	A	A	G	G	C	A	Ins	T	-	A	-	T	G	GT	Ins	Ins	A	CT	G	CAA
SyMattis	H1	T	C	C	A	C	A	T	A	A	G	G	C	A	Ins	T	-	A	-	T	G	GT	Ins	Ins	A	CT	G	CAA
Weebil	H1	T	C	C	A	C	A	T	A	A	G	G	C	A	Ins	T	-	A	-	T	G	GT	Ins	Ins	A	CT	G	CAA
Xi-19	H2	C	T	T	G	A	G	G	G	G	A	C	T	G	Del	C	AG	G	T	C	-	-	Del	Del	-	-	C	TGG

Supplemental Table S5. Alignment of 300kb surrounding *ELF3-D1* in 10+ wheat genome lines. Average nucleotide identity scores from alignment of 300kb sequence surrounding *ELF3-1D* in the 10+ wheat genome cultivars. Alignment produced using the whole genome alignment tool from CLC workbench 21.0.3 using a 15-base seed with no mismatches.

Cultivar	ArinaLrFor	CDC_Landmark	CDC_Stanley	Lancer	Mace	Norin61	CSv1.0	Julius	SYMattis	Jagger	Cadenza
**ArinaLrFor**	100.00	99.82	99.94	99.90	99.95	99.81	99.89	99.93	99.97	91.00	91.44
**CDC_Landmark**	99.82	100.00	100.00	99.89	99.85	99.93	99.82	99.90	99.94	90.57	91.44
**CDC_Stanley**	99.94	100.00	100.00	99.89	99.95	99.97	99.89	99.92	99.94	90.76	91.44
**Lancer**	99.90	99.89	99.89	100.00	99.94	99.92	99.91	99.94	99.93	91.00	91.42
**Mace**	99.95	99.85	99.95	99.94	100.00	99.90	99.86	99.98	99.98	90.51	91.44
**Norin61**	99.81	99.93	99.97	99.92	99.90	100.00	99.95	99.93	99.95	91.01	91.43
**CSv1.0**	99.89	99.82	99.89	99.91	99.86	99.95	100.00	99.93	99.83	91.00	91.44
**Julius**	99.93	99.90	99.92	99.94	99.98	99.93	99.93	100.00	99.99	91.00	91.43
**SYMattis**	99.97	99.94	99.94	99.93	99.98	99.95	99.83	99.99	100.00	91.00	91.43
**Jagger**	91.00	90.57	90.76	91.00	90.51	91.01	91.00	91.00	91.00	100.00	99.94
**Cadenza**	91.44	91.44	91.44	91.42	91.44	91.43	91.44	91.43	91.43	99.94	100.00

Supplemental Table S6. Promoter motif analysis of *TtELF3.* Column A refers to the A sub-genome, B sub-genome and D to the D sub-genome. Motifs and corresponding sequences taken from (Gendron et al., 2012; Huang et al., 2012; Wang et al., 1997).

Motif	Abbreviation	Sequence	A	B	D	Arabidopsis
Evening element	EE	AAAATATCT	0	1	0	0
Evening Like element	ELE	AATATCT	1	0	0	1
CCA1 binding site (A variant)	/	AAAAATCT	0	0	0	0
CCA1 binding site (C variant)	/	AACAATCT	0	0	0	2
Morning element 1	ME 1	CCACAC	0	1	1	0
Morning element 2	ME 2	GTGTGG	1	1	2	0
Hormone Up at Dawn 1	HUD 1	CACATG	2	3	4	2
Hormone Up at Dawn 2	HUD 2	CATGTG	3	9	6	0
G BOX (morning)	/	CACGTG	1	0	3	0
GATA (evening)	/	GGATA	4	8	6	6
Starch box (midnight)	/	AAGCCC	1	0	1	0
Telo-Box (midnight)	/	AAACCCT	2	0	0	0
Protein Box (midnight)	/	ATGGGCC	0	2	1	0

Supplemental Table S7. Promoter motif analysis of *TtLHY.* Column A refers to the A sub-genome, B sub-genome and D to the D sub-genome. Motifs and corresponding sequences taken from (Gendron et al., 2012; Huang et al., 2012; Wang et al., 1997).

Motif	Abbreviation	Sequence	A	B	D	Arabidopsis
Evening element	EE	AAAATATCT	0	0	0	0
Evening Like element	ELE	AATATCT	0	0	0	0
CCA1 binding site (A variant)	/	AAAAATCT	0	1	0	0
CCA1 binding site (C variant)	/	AACAATCT	1	0	0	0
Morning element 1	ME 1	CCACAC	1	1	1	1
Morning element 2	ME 2	GTGTGG	2	0	0	0
Hormone Up at Dawn 1	HUD 1	CACATG	1	1	1	1
Hormone Up at Dawn 2	HUD 2	CATGTG	4	3	3	0
G BOX (morning)	/	CACGTG	1	1	0	1
GATA (evening)	/	GGATA	3	3	3	3
Starch box (midnight)	/	AAGCCC	0	0	1	1
Telo-Box (midnight)	/	AAACCCT	0	0	0	0
Protein Box (midnight)	/	ATGGGCC	0	1	1	0

Supplemental Table S8. Promoter motif analysis of *TtLUX.* Column A refers to the A sub-genome, B sub-genome and D to the D sub-genome. Motifs and corresponding sequences taken from (Gendron et al., 2012; Huang et al., 2012; Wang et al., 1997).

Motif	Abbreviation	Sequence	A	B	D	Arabidopsis
Evening element	EE	AAAATATCT	9	4	4	1
Evening Like element	ELE	AATATCT	3	1	1	2
CCA1 binding site (A variant)	/	AAAAATCT	1	0	0	2
CCA1 binding site (C vairiant)	/	AACAATCT	0	1	0	0
Morning element 1	ME 1	CCACAC	1	1	1	0
Morning element 2	ME 2	GTGTGG	1	1	0	0
Hormone Up at Dawn 1	HUD 1	CACATG	2	1	2	0
Hormone Up at Dawn 2	HUD 2	CATGTG	2	2	0	1
G BOX (morning)	/	CACGTG	2	3	2	2
GATA (evening)	/	GGATA	3	5	2	2
Starch box (midnight)	/	AAGCCC	0	0	2	0
Telo-Box (midnight)	/	AAACCCT	3	2	1	2
Protein Box (midnight)	/	ATGGGCC	1	1	2	0

Supplemental Table S9. Total number of SNPs within the coding sequence of *ELF3* in the 10+ Wheat Genomes project cultivars. A SNP was recorded if the base was different to the Chinese Spring IWGSCRefseqv1.1 sequence in the alignment. SNPs within the same codon were recorded individually. It was not possible to create clear alignments between *ELF3-1D* Chinese Spring, Jagger and Cadenza and so the coding sequence for the three cultivars was downloaded from ENSEMBL plants, aligned and the number of SNPs manually counted. NS = non-synonymous SNP, S= synonymous SNP, PAV = presence/absence variation. Cultivars were considered to share the same allele if the CDS was identical. Zavitan is a tetraploid variety and so lacks a D sub genome.

	**Chromosome**					
	**1A**		**1B**		**1D**	
**Cultivar**	**NS**	**S**	**NS**	**S**	**NS**	**S**
ArinaLrFor	2	2	1	0	0	0
CDC Stanley	2	2	0	0	0	0
CDC Landmark	2	2	0	0	0	0
Cadenza	0	1	1	0	35	23
Claire	0	1	0	0	0	0
Jagger	2	3	0	1	35	23
Julius	PAV	PAV	0	0	1	0
Lancer	2	2	0	0	0	0
Norin61	2	2	0	0	0	0
Mace	2	2	0	0	0	0
PI190962	0	0	0	0	0	0
Paragon	0	1	1	0	0	0
Robigus	0	0	0	0	0	0
SY Mattis	0	1	0	0	0	0
Zavitan	7	2	1	3	*NA*	*NA*
**Total No. SNP's**	21	21	3	4	71	46
**Total No. Alleles**	5		3		3	

Supplemental Table S10. IWGSC RefSeq Annotation v1.1 gene identifiers used for BLAST queries.

	Gene ID			
**Gene Name**	**A homoeologue**	**B homoeologue**	**D homoeologue**	**Undesignated**
ELF3	TraesCS1A02G443200	TraesCS1B02G477400	TraesCS1D02G451200	
FT1	TraesCS7A02G115400	TraesCS7B02G013100	TraesCS7D02G111600	
GI	TraesCS3A02G116300	TraesCS3B02G135400	TraesCS3D02G118200	
LHY	TraesCS7A02G299400	TraesCS7B02G188000	TraesCS7D02G295400	
LUX	TraesCS3A02G526600	TraesCS3B02G594300	TraesCS3D02G531900	
Ppd-1	TraesCS2A02G081900		TraesCS2D02G079600	TraesCSU02G196100
PRR73	TraesCS4A02G105300	TraesCS4B02G198700	TraesCS4D02G199600	
RP15		TraesCS2B02G194500	TraesCS2D02G175800	
RPT5A	TraesCS3A02G421900	TraesCS3B02G457100	TraesCS3D02G417000	
TOC1	TraesCS6A02G227900	TraesCS6B02G253900	TraesCS6D02G207100	
ZTL	TraesCS7A02G431600	TraesCS6B02G426300	TraesCS7D02G423400	
